# Adaptive Therapy Exploits Fitness Deficits in Chemotherapy-Resistant Ovarian Cancer to Achieve Long-Term Tumor Control

**DOI:** 10.1158/0008-5472.CAN-25-0351

**Published:** 2025-04-29

**Authors:** Helen Hockings, Eszter Lakatos, Weini Huang, Maximilian Mossner, Mohammed A. Khan, Nikolina Bakali, Jacqueline McDermott, Kane Smith, Ann‐Marie Baker, Trevor A. Graham, Michelle Lockley

**Affiliations:** 1 Centre for Cancer Cell and Molecular Biology, Barts Cancer Institute, Queen Mary University of London, London, United Kingdom.; 2 Centre for Cancer Evolution, Barts Cancer Institute, Queen Mary University of London, London, United Kingdom.; 3 Department of Mathematical Sciences, Chalmers University of Technology, Gothenburg, Sweden.; 4 Centre for Evolution and Cancer, The Institute of Cancer Research, London, United Kingdom.; 5 School of Mathematical Sciences, Queen Mary University of London, London, United Kingdom.; 6 Pathology Department, Barts Health NHS Trust, London, United Kingdom.

## Abstract

Drug resistance results in poor outcomes for patients with cancer. Adaptive therapy is a potential strategy to address drug resistance that exploits competitive interactions between sensitive and resistant subclones. In this study, we showed that adapting carboplatin dose according to tumor response (adaptive therapy) significantly prolonged survival of murine ovarian cancer models compared with standard carboplatin dosing, without increasing mean daily drug dose or toxicity. Platinum-resistant ovarian cancer cells exhibited diminished fitness when drug was absent *in vitro* and *in vivo*, which caused selective decline of resistant populations due to reduced proliferation and increased apoptosis. Conversely, fitter, sensitive cells regrew when drug was withdrawn. Using a bioinformatics pipeline that exploits copy number changes to quantify the emergence of treatment resistance, analysis of cell‐free DNA obtained longitudinally from patients with ovarian cancer during treatment showed subclonal selection through therapy, and measurements of resistant population growth correlated strongly with disease burden. These preclinical findings pave the way for future clinical testing of personalized adaptive therapy regimens tailored to the evolution of carboplatin resistance in individual patients with ovarian cancer.

**Significance:**

Carboplatin adaptive therapy improves treatment efficacy without increasing daily dose due to reduced fitness of drug-resistant populations, which can be tracked using cfDNA and could direct adaptive therapy in future clinical trials.

*See related commentary by Gatenby, p. 3373*

## Introduction

Systemic cancer treatment is based on the principle that delivery of high drug dose will eradicate all malignant cells and achieve cure. Unfortunately, this paradigm of maximum tolerated dose (MTD) chemotherapy frequently fails, especially in metastatic solid cancers (1). This could be a consequence of therapy selecting for preexisting drug‐resistant subclones (2) or of inherent plasticity in cancer cell phenotypes (3). The paucity of available anticancer drug therapies means that the emergence of a resistant cancer cell population results in treatment failure (1).

Trait evolution is often subject to trade-offs (4); if a cancer clone evolves to become optimal at a particular trait, such as maintaining a resistant phenotype, it may come at the price of being less good at another, for example proliferation (5, 6). It follows that relative fitness of drug‐sensitive and drug-resistant cells is reversed by drug therapy; in the presence of drug, resistant cells are fitter, whereas sensitive cells have higher fitness when drug is absent (7). Similar to other biological systems, fitness costs in cancer are expected to be most apparent in low‐resource settings in which competition for limited resources exposes “suboptimal” phenotypes (5, 8). Adaptive therapy (AT) is a new treatment paradigm that exploits the competitive interactions between sensitive and resistant subclones (6), aiming to maintain a sufficient population of sensitive cells to suppress proliferation of “less fit” resistant cells (9). This approach accepts that within the palliative setting, cancer cannot be eradicated and aims to control rather than cure (7, 10).

High‐grade serous ovarian cancer (HGSC) is the most common ovarian cancer subtype. Standard treatment consists of cytoreductive surgery and combined platinum and taxane chemotherapy (11), followed by PARP inhibitor maintenance in homologous recombination deficient cohorts (12–16). Despite this intensive treatment, most HGSCs recur and patients are subsequently treated with multiple lines of chemotherapy. Platinum agents, cisplatin and carboplatin, form the backbone of treatment and are used repeatedly during the disease course, but at each sequential relapse, they become less effective, ultimately leading to treatment failure (17). Targeted agents and immunotherapies have failed to improve survival and so AT could provide a very helpful strategy for patients with incurable HGSC in whom current MTD treatments have failed. A seminal article demonstrated the benefit of carboplatin AT in mice, (18) but the mechanistic basis and relevance to patients with ovarian cancer have not been elucidated.

AT is predicted to be most beneficial when directed by the size of the emergent resistant population, but platinum resistance mechanisms are poorly defined and are not associated with easily trackable markers such as recurrent single-nucleotide variants (19, 20) or common copy number drivers (21). However, posttreatment HGSCs carry new copy-number alterations (CNA) in addition to their already highly altered genomes that seem to be patient specific (22). We recently developed a bioinformatics pipeline, liquidCNA (LiqCNA; ref. 23), which exploits these CNAs to quantify the emergence of treatment resistance.

Here, we show a significant advantage of carboplatin AT compared with standard dosing in mice with established HGSC, even those with preexisting platinum resistance. We demonstrate that multiple platinum‐resistant HGSC cell lines (evolved *in vitro* and *in vivo*) exhibit reduced fitness in the absence of platinum that is exposed by low-resource conditions. This results in decline of the resistant population, mediated by reduced proliferation and increased apoptosis. We reveal that resistant cell fitness is reversed by platinum treatment such that sensitive/resistant populations fluctuate during drug therapy. Furthermore, in sequential blood samples and biopsies, we demonstrate that LiqCNA measures of the emergent resistant population correlate with tumor progression in HGSC. This crucial development is expected to improve AT by enabling the evolution of therapy resistance to direct adaptive drug dosing in future clinical trials.

## Materials and Methods

### Chemotherapy‐resistant cell lines

**Figure 1.**
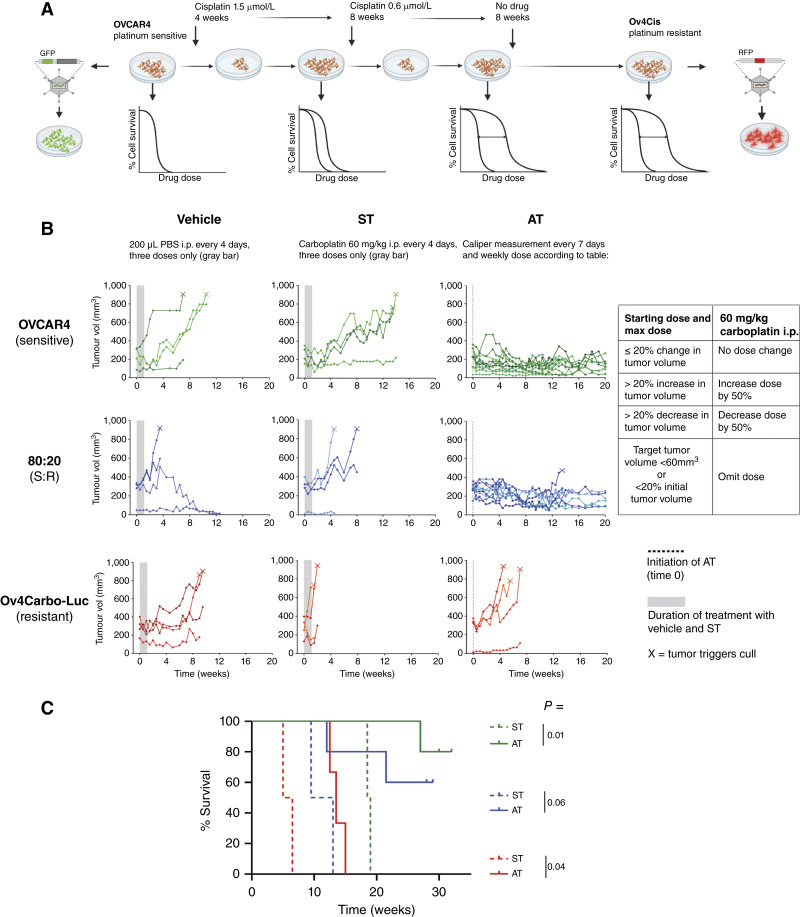
Carboplatin AT significantly extends survival in tumor-bearing mice. **A,** Schematic showing derivation of the resistant cell panel. **B,** Mice were injected subcutaneously in both flanks with 100% OVCAR4 cells (sensitive; green, top row), 80:20 OVCAR4:Ov4Carbo‐Luc cells (blue, middle row), or 100% Ov4Carbo‐Luc cells (resistant; red, bottom row). The same color shade is used for the target and nontarget tumors in the same mouse. Mice were randomized to receive vehicle (i.p. every 4 days x3; left column; *n* = 2 mice per ratio), ST (60 mg/kg carboplatin i.p. every 4 days x3; middle column; *n* = 2 mice per ratio), or AT as per table (right column; *n* = 3–5 mice per ratio). Gray bars, duration of vehicle and ST; dotted line, initiation of AT at time 0. Each line indicates one tumor; X indicates mouse culled. **C,** Kaplan–Meier survival curve for experiment in **B**, measured from the time of subcutaneous tumor cell injection. Green, 100% OVCAR4; blue, 80:20 OVCAR4:Ov4Carbo‐RFP; red, 100% Ov4Carbo‐Luc cells. Dashed lines, ST; solid lines, AT. *P*, significance for the comparisons indicated as assessed by the log-rank test Mantel–Cox.

OVCAR4 (RRID: CVCL_1627) and Cov318 (RRID:CVCL_2419) human HGSC cells were cultured *in vitro* (DMEM, 10% FBS, and 1% penicillin/streptomycin) in cisplatin 0.5 μmol/L, with weekly exchange of media containing cisplatin (0.5 μmol/L) for 8 weeks to create Ov4Cis and Cov-Cis cisplatin-resistant cell lines. Resistance was increased by repeating the process with 0.6 μmol/L cisplatin (Ov4Cis, Fig. 1A) or 0.7 μmol/L cisplatin (Cov-Cis) for another 8 weeks. Carboplatin-resistant OVCAR4 cells (Ov4Carbo) were established by incrementing carboplatin dose every 4 weeks (1.5, 2.0, 3.0, and 3.5 μmol/L). In all cases, drug was then removed for 2 months and maintained resistance was confirmed *in vitro* (Supplementary Table S1; ref. 20).

Cells were lentivirally transfected with firefly luciferase (Luc) and either green fluorescent protein (GFP; sensitive cells) or red fluorescent protein (RFP; resistant cells; ref. 20). We previously generated an additional carboplatin-resistant cell line *in vivo* (IVR01; ref. 20). OVCAR4-GFP cells were injected intraperitoneally (i.p.) and mice with established tumors received four dosages of carboplatin 50 mg/kg i.p., every 7 days. Bioluminescence imaging monitored tumor response. When tumors regrew, mice were culled (15 weeks after initiation of treatment), and tumors were harvested to create IVR01. IVR01 lost GFP expression but *in vitro* carboplatin resistance was comparable with *in vitro*–derived Ov4Carbo cells (20). Resistant cells were immediately expanded without further subcloning, and low-passage cells, cultured without drug, were used in all subsequent experiments with two-weekly *Mycoplasma* testing.

### Cell proliferation and cocultures

Proliferation was quantified using the Incucyte live-cell imaging system (Essen Biosciences). Four images per well were recorded every 4 hours and confluence was analyzed (Incucyte ZOOM 2016B software, RRID: SCR_019874). For coculture studies, GFP-labeled sensitive and RFP-labeled resistant cells were plated in 6 cm dishes (2.5 × 10^5^ total cells) at a range of starting ratios. Cells were then either passaged twice weekly in 10% FBS (standard resource) or 0.5% FBS‐containing media were exchanged every 24 hours (low resource). To measure the effect of cisplatin, 50:50 cocultures were plated in 10% FBS and media were changed to 0.5% FBS after 24 hours as before. Cisplatin (0.1–1 μmol/L) was added on day 6 and media were exchanged with fresh 0.5% FBS‐containing media every 24 hours. Cocultures were harvested over time and total cell number/μL was counted [Countess IIR automated cell counter (Life Technologies)]. GFP expression in DAPI-stained live cells was measured by flow cytometry (BD LSRFortessa; FlowJo v8). Resistant cell abundance was calculated using RFP-expressing cells or by subtracting GFP+ cells from the total. Cell cycle and apoptosis were assessed by PI/RNaseA staining and Annexin V labeling (BV605 BD Horizon, RRID: AB_2869539), respectively, with 100 μmol/L etoposide‐treated cells as positive control in apoptosis assays.

### Growth dynamics modeling

Growth rates and carrying capacities were calculated using Mathematica v.11 and PopDynamics. The effect of the seeding ratio was modeled by using the lowest sensitive seeding ratio (5:95 and 15:85 for high and low resource, respectively) to fit the log [sensitive:resistant (S:R)] ratios over time using a linear model. The slope of this fitted line measures g = gs − gr. Datasets obtained at other seeding ratios were aligned so that the first time‐point of each dataset fell on the line.

### Conditioned media assay

In six‐well plates, 2.5 × 10^5^ OVCAR4‐GFP or Ov4Cis cells were seeded in 10% FBS, and daily media replacement was done with 0.5% FBS‐containing medium preconditioned for 24 hours by either the same cell line, the opposite cell line, or a 50:50 OVCAR4‐GFP:OV4Cis‐RFP coculture. Cells were harvested and counted (Countess IIR, Life Technologies).

### PCR

DNA was extracted using the DNeasy Kit (Qiagen) following homogenization and lysis of tissue samples. GFP and RFP DNA was quantified by qPCR (QuantStudio 5; Applied Biosystems), normalized to human GAPDH.

GFP, forward: 5′GGA​CGA​CGG​CAA​CTA​CAA​GA‐3′ and reverse: 5′‐TTG​TAC​TCC​AGC​TTG​TGC​CC‐3′

RFP, forward: 5′‐TGGTGTAG TCCTCGTTGTGG‐3′ and reverse: 5′‐ATG​AGG​CTG​AAG​CTG​AAG​GA‐3′

Human GAPDH, forward: 5′‐CCTCACAGTTGCC ATGTAGACC‐3′ and reverse: 5′‐TCA​GTC​TGA​GGA​GAA​CAT​ACC​A‐3′.

mRNA was quantified by RT-PCR normalized to β‐actin with doxorubicin‐treated cells (100 ng/mL) as positive controls.

*CDKN2a *(p16), forward: 5′CAA​CGC​ACC​GAA​TAG​TTA​CG‐3′ and reverse: 5′‐CAG​CTC​CTC​AGC​CAG​GTC‐3′

*CKDN1a* (p21), forward: 5′‐ GGC​AAG​AGT​GCC​TTG​ACG​AT‐3′ and reverse: 5′‐ CCT​CTT​GAC​CTG​CTG​TGT​CG‐3′

β‐actin: forward: 5′‐AGA​GCT​ACG​AGC​TGC​CTG​AC‐3′ and reverse: 5′CGT​GGA​TGC​CAC​AGG​ACT‐3′.

### Animal studies

Experiments were conducted under the UK Home Office project license P1EE3ECB4 and were approved by the Institutional Review Board. Six-week-old female CD1^nu/nu^ mice (Charles River Laboratories, RRID: IMSR_CRL:086) were injected intraperitoneally or subcutaneously in both flanks with 5 × 10^6^ cells in 200 μL sterile PBS. Tumors were measured using calipers [volume = π (short diameter)^2^ × (long diameter)/6]. Animals were killed via a Schedule 1 method when the volume of either flank tumor reached 1.44 cm^2^, at experimental endpoint, or if the project license’s maximum severity was reached.

### AT experiment

Inclusion criteria included mice with one subcutaneous tumor (target tumor) ≥300 mm^3^. Mice were randomly allocated to (i) vehicle (200 μL PBS i.p. every 4 days x3 doses); (ii) standard therapy (60 mg/kg carboplatin i.p. every 4 days x3 doses); and (iii) AT consisting of one initial dose 60 mg/kg carboplatin i.p., then weekly i.p. carboplatin (see Fig. 1B). Nontarget tumor sizes were never used to determine drug dosage. Mice were monitored daily and weighed weekly. If either flank tumor (target or nontarget) reached 1.44 cm^2^ or mice lost >15% starting body weight, animals were killed via a Schedule 1 method in accordance with our license.

### IHC

Tumors were fixed in 4% paraformaldehyde. Sections (4 μm) were dewaxed, rehydrated, treated with 3% H_2_O_2_, and subjected to antigen retrieval (Tris-EDTA, pH 9.0, at 95°C). Blocking was done with PBS + 5% goat serum and 1% BSA (1 hour at room temperature). Primary antibodies (1 hour at room temperature): p53 (Cell Signaling Technology, 2527, RRID: AB_10695803; 1:200), GFP (Cell Signaling Technology, 2956, RRID: AB_1196615; 1:75), anti–cleaved caspase‐3 (Cell Signaling Technology, 9664, RRID: AB_2070042; 1:50), and luciferase (Abcam, ab185924, RRID: AB_2938620; 1:200). Secondary biotinylated anti‐rabbit antibody and streptavidin‐peroxidase were applied (at room temperature for 45 minutes), followed by hematoxylin counterstaining. Slides were digitized (Hamamatsu NanoZoomer‐XR). p53+ and GFP+ pixels were quantified using Adobe Photoshop (RRID: SCR_014199).

### Patient samples

Tumor tissue and blood were collected from patients with stage III/IV HGSC. All patients provided written informed consent under the ethics of the Barts Gynae Tissue Bank (REC: 20/EE/0193), in accordance with the Declaration of Helsinki and following approval by an institutional review board. Blood was collected in 10 mL Cell‐Free DNA BCT (Streck) tubes and centrifuged within 4 hours (1,200 × *g* for 10 minutes at 40°C); the supernatant was stored at −80°C for cell-free DNA (cfDNA) extraction. Cell (leukocyte) pellets were stored at −80°C for germline DNA extraction.

### DNA extraction and analysis

Leukocyte pellets were treated with red blood cell lysis buffer (distilled water containing 155 mmol/L NH_4_Cl, 10 mmol/L KHCO_3_, and 1 mmol/L EDTA), and genomic DNA was extracted [DNeasy Blood and Tissue Kit (Qiagen)]. DNA was extracted from formalin‐fixed, paraffin‐embedded (FFPE) tissue [High Pure FFPET DNA Isolation Kit (Roche)] following laser capture microdissection. Genomic and tissue DNA was fragmented (Covaris M220), and libraries were prepared using the NEBNext FFPE DNA Repair Mix and NEBNext Ultra II DNA Library Prep Kit for Illumina (New England Biolabs Inc). cfDNA extraction and library preparation used the QIAseq cfDNA All‐in‐One Kit (QIAGEN). Libraries were sequenced with Illumina NovaSeq 6000 (average depth: 0.3× leucocytes, 0.5× tissue, and 1.9× cfDNA). Reads were aligned to hg19. Copy number profiles were obtained using QDNAseq (24) and analyzed with LiqCNA as previously described (23) to obtain tumor purity and estimate the resistant population. LiqCNA was run 150 times per patient on a random 75% subsample of genomic segments to derive 95% confidence intervals for each subclonal ratio estimate.

### Statistical analysis

Analysis was performed using GraphPad Prism v7.04 (RRID: SCR_002798). Significance levels were *, *P* < 0.05; **, *P* < 0.001; ***, *P* < 0.0001. Pearson R and *R*^2^ were used to measure linear correlation and fit. Paired, two‐tailed *t* tests were used unless otherwise stated.

### Data availability

The genomics data generated in this study are publicly available in the European Genome-Phenome Archive (EGA) at EGAS50000001142. The copy number profiles and liquidCNA algorithm output for patient samples are publicly available at https://doi.org/10.17632/m93sk9n767.1 or https://data.mendeley.com/datasets/m93sk9n767/1. All other raw data are available upon request from the corresponding authors.

## Results

### Establishing a platinum-resistant HGSC cell line panel

Platinum-resistant HGSC cells were evolved from two cell lines (OVCAR4 and Cov318) that reflect the genomic features of human HGSC (Fig. 1A; Supplementary Table S1; ref. 25). We previously demonstrated that these platinum-resistant cell lines evolved from a preexisting ancestral clone (20), and in common with human HGSC (22, 26), they do not share a mutational cause of drug resistance (20). They have however evolved recurrent gene expression changes that significantly overlap with three independent datasets from relapsed patients with HGSC (20). Sensitive cells were transfected with firefly luciferase (Luc) and either GFP (sensitive cells) or RFP (resistant cells) as previously described (20).

### Carboplatin AT significantly extends survival in tumor-bearing mice

To test AT *in vivo* (Fig. 1B), we used ancestral OVCAR4 cells (platinum sensitive) and Ov4Carbo (carboplatin resistant) as both cell lines form tumors in mice (20). Moreover, carboplatin is the most commonly used drug in ovarian cancer care. In a pilot experiment, we demonstrated that mean subcutaneous tumor growth was comparable in OVCAR4 and Ov4Carbo cells although growth was variable between individual tumors (Supplementary Fig. S1). Ov4Carbo (but not OVCAR4) were transfected with RFP/firefly luciferase (Ov4Carbo-Luc) to enable tracking of the resistant population over time using bioluminescent imaging. Subcutaneous tumors were grown in both flanks of female nude mice using either OVCAR4 cells (sensitive), Ov4Carbo‐Luc (resistant), or an 80:20 coculture of OVCAR4:Ov4Carbo‐Luc. Mice were monitored and treated as described in Materials and Methods. Standard carboplatin treatment (ST) was the same regimen as the seminal AT article by Gatenby and colleagues (ref. 18; 60 mg/kg carboplatin i.p. every 4 days for three doses). Our AT regimen was based on previous studies (18, 27) and was administered intraperitoneally weekly according to changes in tumor size (Fig. 1B, Table). Both target and nontarget tumors are shown (Fig. 1B), with the same color shade being used for the two tumors in the same mouse. Median survival was calculated from the time of initial tumor cell injection (Fig. 1C).

All mice treated with vehicle and ST reached humane endpoint before the end of the experiment (marked X, Fig. 1B). ST temporarily halted tumor growth in mice with majority sensitive tumors (OVCAR4 and 80:20 OVCAR4:Ov4Carbo‐Luc) but all tumors re‐grew once treatment stopped, and there was no difference in survival between vehicle and ST in these two tumor groups. Conversely, in mice with Ov4Carbo‐Luc tumors (resistant), median survival following ST was 5.75 weeks compared with 15.25 weeks with vehicle (*P* = 0.09, log-rank test, Mantel–Cox).

AT improved survival compared with ST (Fig. 1C). In mice with Ov4Carbo‐Luc tumors (resistant), AT increased survival compared with ST (*P* = 0.04) but all mice still progressed during AT and reached humane endpoint by week 8. In mice with OVCAR4 tumors (sensitive), median survival following AT was undefined because there were too few deaths in this group, compared with 18.75 weeks with ST (*P* = 0.01). One mouse in the OVCAR4 AT‐treated group was culled 15 weeks after treatment initiation because of unexplained weight loss with no tumor identified at necropsy. The other four mice survived without tumor growth until experimental end point (20 weeks). In mice with 80:20 OVCAR4:Ov4Carbo‐Luc tumors, AT also achieved durable tumor control such that median survival could not be defined, compared with median survival of 11.25 weeks following ST (*P* = 0.06).

**Figure 2.**
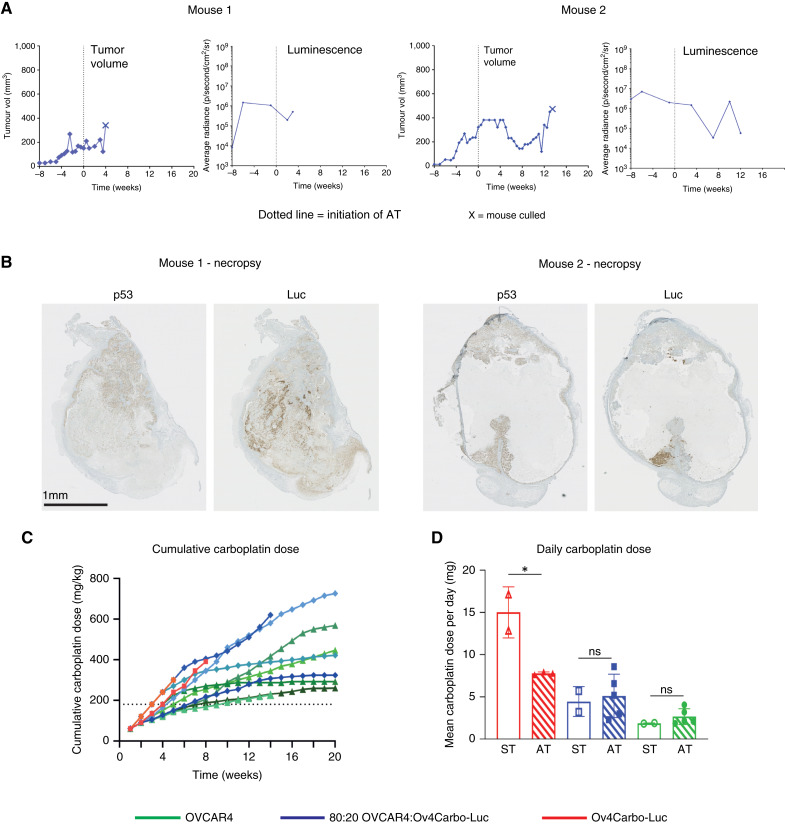
Dynamics of resistant cell growth *in vivo* in relation to carboplatin dose. **A,** Tumor volume (vol) and bioluminescence of subcutaneous tumors over time in AT-treated mice bearing 80:20 OVCAR4:Ov4Carbo‐Luc tumors that were culled at 4 weeks (mouse 1; left) and 13.5 weeks (mouse 2; right) after initiation of treatment (dotted line) because of large, hemorrhagic tumors. **B,** IHC for p53 (tumor cells) and firefly luciferase (resistant population) in tumors shown in **A**. **C,** Cumulative dose of carboplatin in mg/kg over time for all mice receiving AT. Green, OVCAR4; blue, 80:20 OVCAR4:Ov4Carbo-Luc; red, Ov4Carbo-Luc, with a different shade for each mouse. Dashed line indicates cumulative carboplatin dose ST. **D,** Carboplatin dose per day according to the injected cell ratio and treatment group. Data are shown as mean ± SD, with *n* = 2 to 5 mice per group. ns, nonsignificant; *, *P* < 0.05, paired *t* test.

Two AT-treated mice with 80:20 tumors reached home office limits before experimental endpoint (four and 13.5 weeks after treatment initiation) because of large, hemorrhagic tumors. In both cases, this was associated with increased light output (bioluminescent imaging) compared with nadir although light output had decreased again in the second of the two mice on the day of death (13.5 weeks; Fig. 2A). Histology revealed that the mouse that died at 4 weeks had a tumor that was dominated by resistant, luciferase‐expressing cells. In the mouse that died at 13.5 weeks, the enlarged tumor was cystic with a central necrotic core, which may explain the reduced light output at end point (Fig. 2B).

All AT‐treated mice received a higher cumulative carboplatin dosage than ST mice (Fig. 2C). In mice with OVCAR4 and 80:20 tumors, total carboplatin dosage plateaued over time as tumor size was controlled (Supplementary Fig. S2A and S2B), such that at later time points, weekly carboplatin was either omitted completely (three mice) or repeatedly administered at very low dose (<3 mg/kg, six mice) in accordance with our AT protocol (Fig. 1B, Table). Although resistant tumors (Ov4Carbo-Luc) still grew despite this increased cumulative carboplatin (Supplementary Fig. S2C), prolonged survival with AT meant that total carboplatin dose per day was significantly lower in AT compared with ST (Fig. 2D; Supplementary Fig. S2D). In mice with OVCAR4 and 80:20 tumors, there was no significant difference in carboplatin dose per day between the two treatment groups (Fig. 2D). Animal weights were comparable between ST and AT (Supplementary Fig. S3A and S3B). The only potential treatment-related toxicity was unexplained weight loss resulting in early death in one of 13 AT-treated animals (described above and Supplementary Fig. S3A, asterisk). AT was therefore well tolerated and significantly improved survival compared with ST, without increasing mean daily carboplatin exposure.

### Drug‐resistant HGSC populations exhibit reduced fitness in low-resource conditions

**Figure 3.**
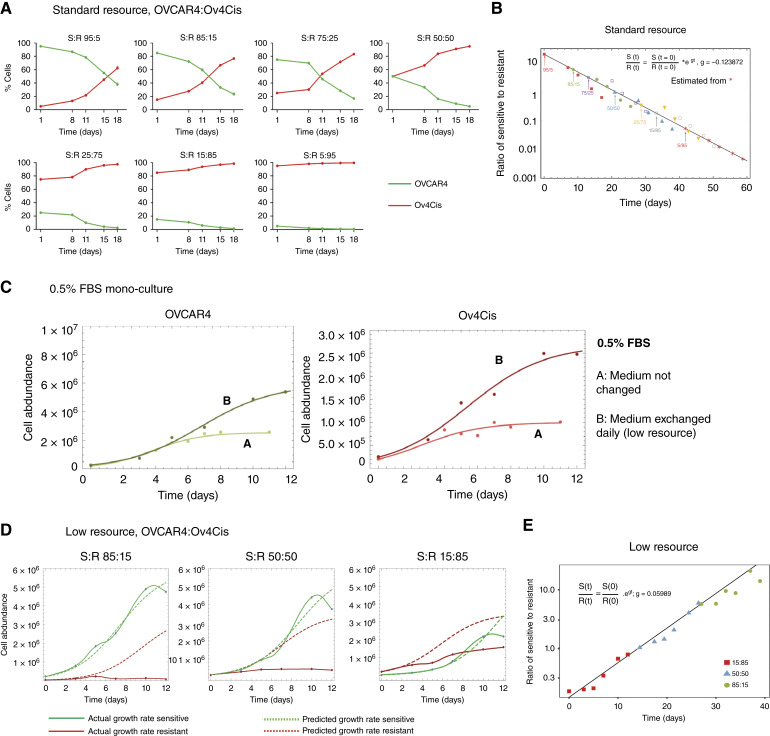
Drug-resistant HGSC exhibits reduced proliferative fitness in low-resource conditions. **A,** Abundance of sensitive (OVCAR4) and resistant (Ov4Cis) cells cocultured over time in high-resource conditions (10% FBS) at a range of starting ratios and passaged every 3 days. Green, OVCAR4; red, Ov4Cis. Data are shown as mean ± SD, with *n* = 3 technical replicates. **B,** Ratio of OVCAR4:OV4Cis in **A** plotted on a log scale over time with start times staggered (so day 0 of the 85:15 ratio experiment is plotted at the time point when there were 85% sensitive cells remaining in the 95:5 starting ratio experiment). The black line shows a linear fit of this log ratio based on the 5:95 dataset (red stars). The slope of the line corresponds to the difference in growth rate between sensitive and resistant cells: g_s_ − g_r_ = g. **C,** Sensitive OVCAR4 cells (green; left) and resistant Ov4Cis (red; right) cells were grown as monocultures in 0.5% FBS-containing media that were either not changed (A) or exchanged daily with fresh 0.5% FBS-containing media (B). Mean cell abundance is shown over time. **D,** Abundance of sensitive (OVCAR4) and resistant (Ov4Cis) cells over time when cocultured without passage in low-resource conditions (0.5% FBS exchanged daily) at three starting ratios (85% sensitive, 50% sensitive, and 15% sensitive). Solid lines indicate measured abundance of sensitive (green) and resistant (red) populations over time. Dashed lines indicate predicted cell growth based on their initial seeding density and their measured growth as monocultures. Data are shown as mean ± SD. Model is based on three technical repeat experiments. **E,** Ratio of OVCAR4:OV4Cis in **D** plotted on a log scale over time. The black line shows a linear fit of this log ratio based on the 85:15 dataset.

To explore the hypothesis that resistant cancer cells have reduced fitness, we created *in vitro* cocultures by seeding OVCAR4 (sensitive) together with Ov4Cis (resistant) in 10% FBS, passaged twice weekly (“Standard Resource”) at different S:R ratios. Cocultures were harvested over time, and sensitive/resistant populations were quantified as described in Materials and Methods. In all cases, the resistant cell population increased over time (Fig. 3A). The rate of decrease in S:R cell ratio was similar for all seeding ratios, with all data points lying close to the line of best fit (Fig. 3B). This gives strong evidence of independent growth without competition in standard resource conditions.

As trade-offs are expected when resources are constrained (5, 8), we tested growth in media containing 0.5% serum. When cells were maintained without media change, there was no difference in the initial growth rate between 0.5% and standard 10% serum (Supplementary Fig. S4). We then compared long-term culture without passage in media containing 0.5% serum either without media change (Fig. 3C:A) or with daily exchange of fresh 0.5% serum-containing media (Fig. 3C:B). OVCAR4 and Ov4Cis cells entered logistic growth in both serum conditions; however, when cells were maintained without media change, abundance of both cell lines reduced rapidly after day 11. Daily media change permitted a faster growth rate, higher carrying capacity, and prolonged survival for both cell lines (Fig. 3C). Culture in 0.5% FBS with daily media exchange was therefore used in all subsequent *in vitro* experiments (“Low Resource”) to maintain cocultures over a longer experimental period.

OVCAR4 and Ov4Cis were then cocultured in low-resource conditions at three starting ratios (Fig. 3D). For all ratios, the presence of resistant cells did not affect sensitive cell growth, but the presence of sensitive cells slowed the growth and lowered the abundance of resistant cells compared with monoculture. In these low-resource conditions, growth rates of mixed sensitive/resistant populations were logistic and deviated from expected growth at early time points, indicating that populations were competing for resources although carrying capacity constrained total population size later in the experiment. Further analysis demonstrated that resistant cell growth rates were lower than those for sensitive cells (*g*_s_ − *g*_r_ = 0.08 doublings/day) and remained independent of the initial ratio of S:R cells (Fig. 3E). This demonstrates the fitness cost borne by drug-resistant cells in low-resource conditions and shows that competition for limited resources penalizes resistant cancer cells.

**Figure 4.**
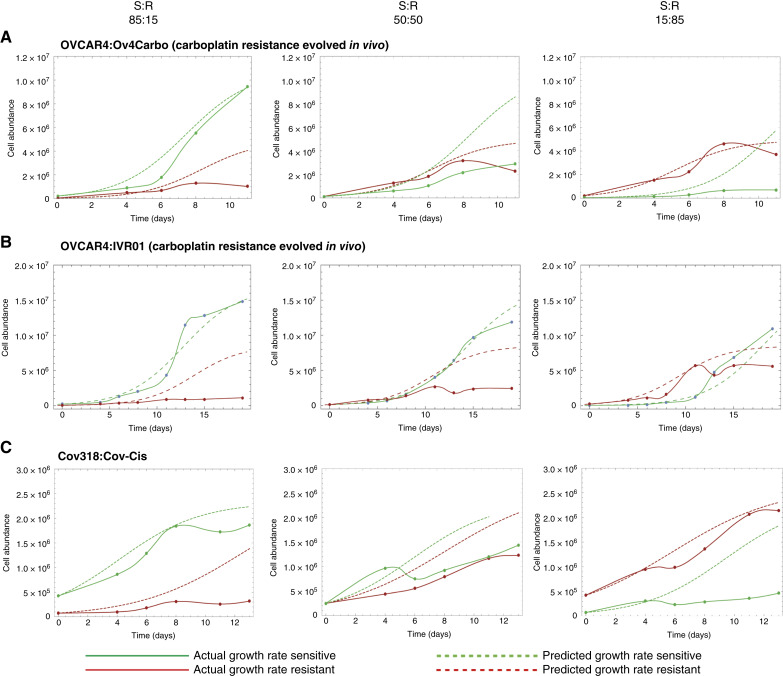
Fitness costs are observed in multiple platinum-resistant HGSC cell lines Abundance of three different sensitive (green) and resistant cell pairs (red) over time when grown as cocultures in low-resource conditions (0.5% FBS exchanged daily) at three starting ratios (85% sensitive, 50% sensitive, and 15% sensitive). **A,** Ov4Carbo: resistance evolved from OVCAR4 *in vitro*. **B,** IVR01: resistance evolved from OVCAR4 *in vivo.***C,** Cov-Cis: resistance evolved from Cov318 *in vitro.* Solid lines indicate observed cell growth and dashed lines indicate predicted cell growth based on 100% monoculture experiments. Models are derived from three technical repeats.

Low-resource cocultures were repeated in three more S:R HGSC cell pairs: OVCAR4:Ov4Carbo, OVCAR4:IVR01, and Cov318:Cov-Cis (Fig. 4). Growth and abundance of the two carboplatin-resistant cell lines, Ov4Carbo (evolved *in vitro*; Fig. 4A) and IVR01 (evolved *in vivo*; Fig. 4B), was reduced by the presence of sensitive cells with stronger competition at carrying capacity. In the Cov318:Cov-Cis pair, growth rates and carrying capacities were comparable in mono‐ and coculture and competition always had a larger impact on the less abundant cell line (Fig. 4C).

### Coculture induces apoptosis in resistant HGSC cells

**Figure 5.**
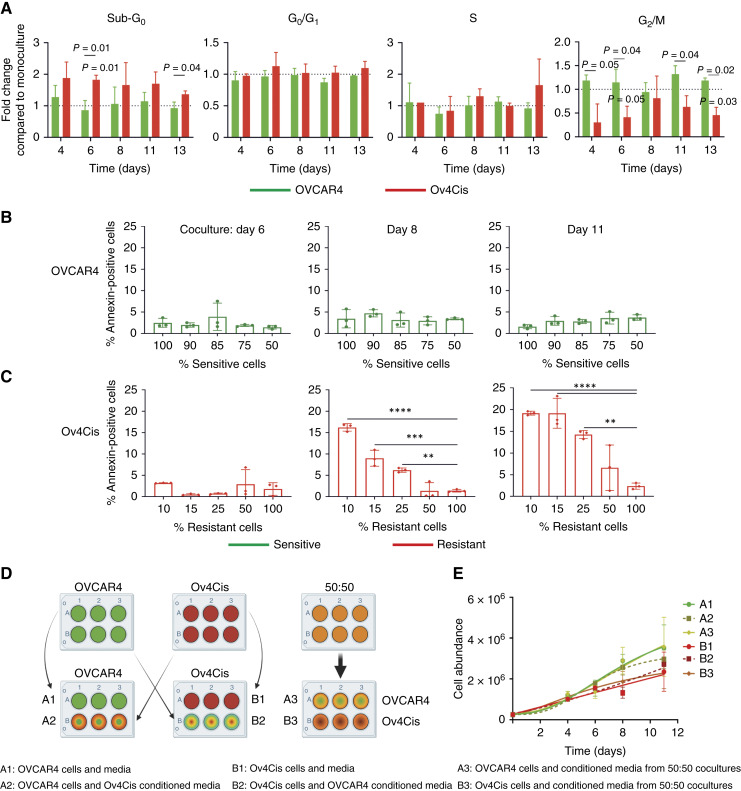
Competition induces apoptosis in less fit, resistant HGSC cells. **A,** Cell cycle profiles of OVCAR4 and Ov4Cis cells grown in coculture at a starting ratio of 85:15 OVCAR4:Ov4Cis in low-resource conditions (0.5% FBS exchanged daily). Cells are normalized to the same cell type in monoculture at the same time point. Data are shown as mean ± SD, with *n* = 3 biological replicates, assessed by a paired *t* test. *P* = OVCAR4 compared with Ov4Cis; *P* = significance between the same cell line in coculture and monoculture at the indicated time point. **B **and** C,** OVCAR4 (**B**) and Ov4Cis (**C**) cells were grown in low-resource coculture at different starting ratios: 100:0, 90:10, 85:15, 75:25, 50:50, and 0:100 and stained with propidium iodide and Annexin V over time. Cocultures were sorted into green fluorescent (sensitive) and non‐fluorescent (resistant) populations and Annexin V positivity was measured by flow cytometry. Data are shown as mean ± SD, with *n* = 3 biological replicates, assessed by a one‐way ANOVA compared with 100% control samples. **, *P* < 0.01; ***, *P* < 0.001; ****, *P* < 0.0001. **D,** OVCAR4 (sensitive; green), Ov4Cis (resistant; red), and OVACR4:Ov4Cis 50:50 cocultures (orange) were grown in low-resource conditions. Every 24 hours, media were exchanged for media that had been preconditioned for 24 hours by either the same cell line (A1 and B1), the other cell line (A2 and B2), or a 50:50 OVCAR4:Ov4Cis coculture (A3 and B3). **E,** Cell abundance over time (mean ± SD, with *n* = 3 technical replicates).

To examine the mechanisms by which resistant populations decline in low-resource cocultures, OVCAR4 (sensitive) and Ov4Cis (resistant) cells were grown as 100% monocultures or 85%:15% S:R cocultures, and cell cycle profiles were obtained by FACS for up to 13 days (Fig. 5A). There was no significant change in the proportion of OVCAR4 cells in any phase of the cell cycle in coculture compared with monoculture. In contrast, compared with Ov4Cis monoculture, more Ov4Cis cells in coculture seemed to be in sub‐G_0_ and fewer Ov4Cis were in the G_2_–M phase. When compared with OVCAR4, there were also more Ov4Cis cells in sub‐G_0_ and fewer Ov4Cis cells in G_2_. Together, this implies that Ov4Cis cells undergo apoptosis and reduced proliferation when cocultured with OVCAR4 cells in low resources.

To further characterize apoptosis, low-resource cocultures were repeated by adding the standard apoptotic marker, Annexin V. Annexin V was comparable in OVCAR4 and Ov4Cis monocultures and did not change over time. In OVCAR4 cells, Annexin V staining was not affected by the presence of resistant cells (Fig. 5B). In contrast, in Ov4Cis cells, Annexin V progressively increased as the size of the sensitive population increased (Fig. 5C). This was most marked at later time points, indicating increased apoptosis of resistant cells over time in low-resource coculture.

OVCAR4 and Ov4Cis cells were then grown either as 100% monocultures or 50:50 cocultures in low-resource conditions. Media were replaced daily as before with conditioned medium obtained either from the same cell line, the other cell line, or the 50:50 coculture (Fig. 5D). Growth of OVCAR4 again exceeded that of Ov4Cis but conditioned media did not influence growth rate or carrying capacities for any of the cell cultures, implying that reduced growth of resistant cell populations in coculture is not induced by secreted factors (Fig. 5E). To investigate senescence as a possible cause of reduced resistant cell population growth, we again seeded cocultures in low-resource conditions and measured the standard senescence markers p16 and p21 by qRT‐PCR. Although doxorubicin induced p21 in Ov4Cis cells (Supplementary Fig. S5A), low-resource coculture failed to induce p16 (Supplementary Fig. S5B) or p21 (Supplementary Fig. S5C) in either cell line for up to 13 days.

### Resistant cells proliferate less when cocultured with sensitive cells *in vivo*

**Figure 6.**
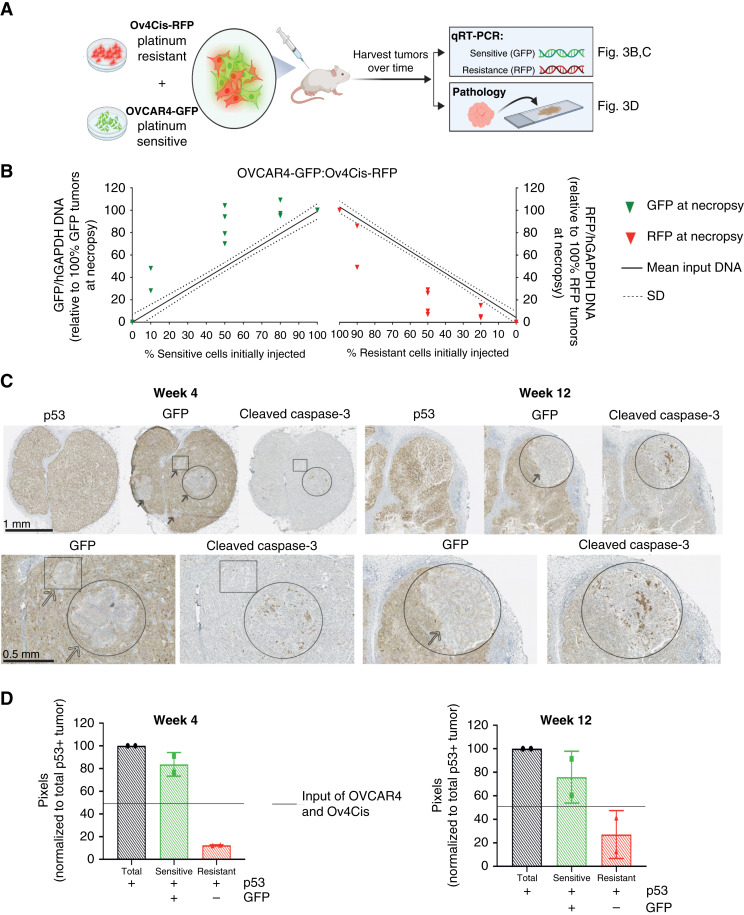
Fitness costs of resistance are observed *in vivo*. **A,** Schematic depicting *in vivo* coculture experiments. **B,** qPCR of GFP and RFP DNA in subcutaneous coculture tumors (OVCAR4:Ov4Cis, 0:100, 10:90, 50:50, 80:20, and 100:0). Tumors were harvested after 12 weeks and qPCR for GFP and RFP is plotted against a standard curve of input DNA (see Supplementary Fig. S7). Solid line, mean; dashed line, SD, one icon per tumor; and *n* = 2 to 4 tumors per starting ratio. **C,** IHC for p53, GFP, and cleaved caspase‐3 in 50:50 OVCAR4:Ov4Cis tumors harvested from different mice at weeks 4 and 12. Representative slides are shown at low (top) and high (bottom) magnification. Arrows indicate p53+, GFP-negative resistant cells. Circles, cleaved caspase-3–positive resistant cells; squares, cleaved caspase-3–negative resistant cells. **D,** Pixel quantification of sensitive and resistant cells. Data are shown as mean ± SD, with *n* = 2 mice per time point. Solid line indicates the starting ratio of S:R cells.

Next, competition between sensitive/resistant populations was characterized *in vivo*. Due to the longer time course of *in vivo* compared with *in vitro* experiments, we first measured GFP and RFP expression by FACS during serial passage *in vitro.* Although GFP fluorescence was preserved for 20 passages, RFP fluorescence diminished over time (Supplementary Fig. S6). To address this, we used qPCR to quantify GFP/RFP in cocultured cells and observed close correlation between qPCR and the known input value of OVCAR4 (S; Supplementary Fig. S7, i) and Ov4Cis (R; Supplementary Fig. S7, ii) cells (*R*^2^ = 0.99). OVCAR4 and Ov4Cis cells were then mixed and subcutaneous tumors were created by injecting mice with the cell mixture in both flanks (Fig. 6A). Mice were culled at 12 weeks, and tumor DNA was extracted from subcutaneous coculture tumors. GFP (sensitive) and RFP (resistant) were quantified by qPCR and plotted against the standard curve as shown in Supplementary Fig. S7. In all tumors, the sensitive population at end point exceeded the initial injected ratio, whereas the resistant population was lower than the starting ratio (Fig. 6B), indicating preferential growth of sensitive cells *in vivo* without drug treatment.

To characterize the temporal dynamics of sensitive/resistant populations *in vivo*, mice were injected subcutaneously in both flanks with mixtures of OVCAR4 and Ov4Cis at a starting ratio of either 50:50 or 80:20. Four mice in each group were culled at weeks 4, 8, and 12. The ratio of S:R cells was quantified by qPCR in the pre-inoculation cell mixture and in the tumors harvested at end point. Again, at all time points and both starting ratios, GFP DNA increased and RFP DNA decreased compared with the input ratio (time = 0; Supplementary Fig. S8).

The contralateral flank tumors from mice with 50:50 OVCAR4‐GFP:Ov4Cis‐RFP tumors in Supplementary Fig. S8 were stained by IHC for p53 to indicate tumor and for GFP to indicate platinum-sensitive tumor cells. As RFP (but not GFP) protein expression was lost over time (Supplementary Fig. S6), resistant Ov4Cis cells were indicated by p53+, GFP-negative staining. Small, discrete islands of GFP‐negative resistant cells were observed embedded within GFP+ tumor nodules (Fig. 6C). This spatial contiguity of resistant cells implied that they were a clonal expansion, rather than a conglomerate of surviving cells. Moreover, of 11 GFP-negative clusters identified in the five tumor nodules examined, only one was located at the tumor periphery (Fig. 6C, week 12) and only one was adjacent to an area of necrosis, supporting our earlier findings that resistant cells are less fit in low-resource conditions. In keeping with our *in vitro* data, positivity for the apoptotic marker cleaved caspase‐3 was most apparent in resistant, GFP‐negative cells (Fig. 6C, circles) although this was not always the case (Fig. 6C, week 4, square). Consistent with our other findings (Fig. 6B; Supplementary Fig. S8), quantification of sensitive and resistant populations again demonstrated that the GFP‐positive sensitive population increased over time and exceeded the 50% initially injected, whereas the GFP‐negative resistant population declined (Fig. 6D).

### Sensitive and resistant HGSC populations grow and decline dynamically during treatment

**Figure 7.**
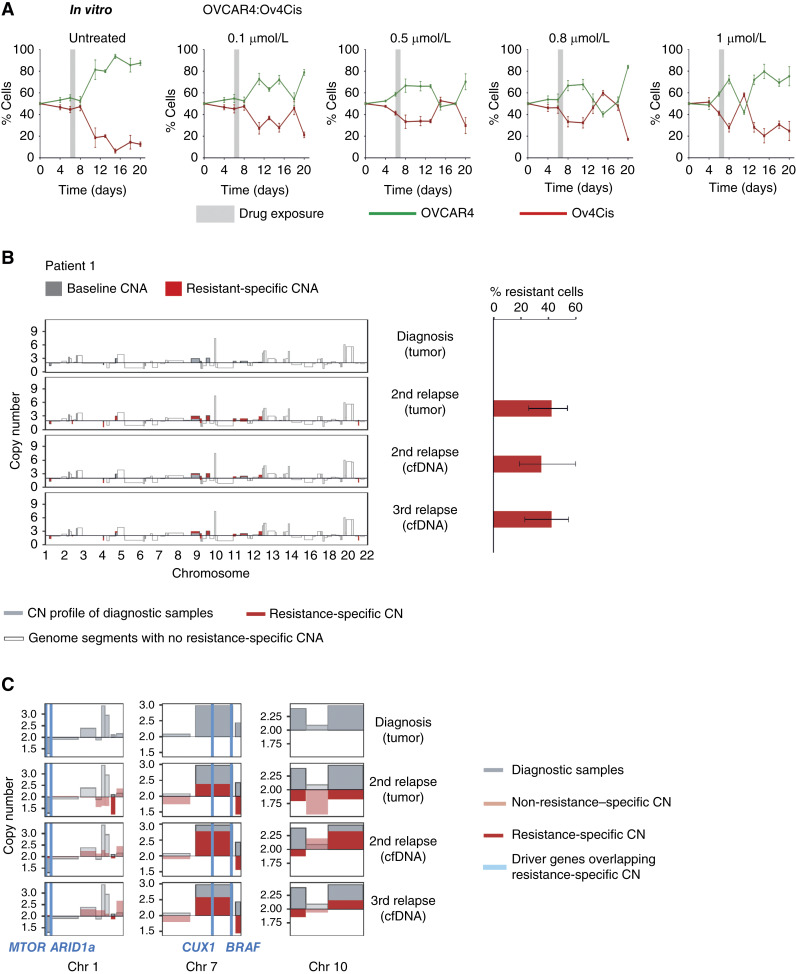
Sensitive and resistant HGSC populations grow and decline dynamically during treatment and can be tracked in cfDNA from patients with HGSC. **A,** OVCAR4 and Ov4Cis cells were grown as 50:50 cocultures in low-resource conditions. On day 6, cells were treated with cisplatin (0.1–1 μmol/L) or vehicle (dashed line). Cultures were harvested over time and the ratio of sensitive (green) to resistant (red) cells was measured by flow cytometry over time. Data are shown as mean ± SD, with *n* = 3 technical replicates. **B,** Tumor purity–corrected copy number profile for each sample obtained from Patient 1. Gray bars show the copy number (CN) profile of diagnostic samples, and red bars show CN profile of later samples as indicated. Transparent bars show genome segments with no resistance‐specific CNAs (i.e., regions with clonally shared CN or with other CNAs). Resistant proportion of each sample estimated by LiqCNA is shown. Error bars indicate the 95% confidence interval of each estimate. **C,** Zoomed‐in profiles of selected chromosomes with the most prominent/impactful resistance‐specific CNAs. Driver genes or genes associated with ovarian cancer that overlap with resistance‐specific CNA are indicated by blue vertical lines and listed below each graph.

To track the influence of chemotherapy on sensitive/resistant populations, 50:50 cocultures of OVCAR4 (sensitive):Ov4Cis (resistant) cells were grown in low-resource conditions *in vitro*. A single dose of cisplatin (0–1 μmol/L) was administered on day 6 and washed off after 24 hours during the next scheduled exchange of low-serum media. The proportion of S:R cells was measured over time. As before, sensitive cell growth exceeded that of resistant cells, with minor biological variability in pretreatment growth rates between the five experiments (Fig. 7A). Drug treatment always reduced the proportion of sensitive cells several days after exposure (range: 5–12 days). This relationship was dependent on dose, such that a greater reduction in the sensitive population was seen at earlier time points with higher cisplatin dose. In all cases, sensitive cells subsequently outgrew resistant ones, presumably as drug effect wore off, demonstrating that the relative size of sensitive/resistant populations changes dynamically during treatment.

### Carboplatin-resistant populations can be tracked in cfDNA from patients with HGSC

Circulating tumor markers, such as CA125 in HGSC, estimate total tumor burden but AT is predicted to be most effective when directed by resistant population growth. Currently, there are no biomarkers to estimate carboplatin resistance. To address this, we applied our bioinformatics pipeline, LiqCNA, to sequential blood and tissue samples from five patients with HGSC during ST. LiqCNA identifies CNAs present in an emerging resistant subclone and infers the frequency of that subclone (23). Three of these five patients were sampled at ≥3 time points (Patients 1, 2, and 3), and two patients were sampled at two time points (Patients 4 and 5). We note that from only two samples, LiqCNA could not reliably distinguish between pervasive ongoing copy number instability and measurement biases from CNAs present exclusively in a subclone. Therefore, the subclonal ratio may have been overestimated in Patients 4 and 5.

CNA profiles showed that resistance‐specific changes emerged through therapy (Patient 1, Fig. 7B; Patients 2–5, Supplementary Fig. S9A, i–S9D, i) and enabled quantification of an emergent resistant population in all five cases (Fig. 7B; Supplementary Fig. S9A, ii–S9D, ii). The most prominent CNAs in the emergent resistant population are shown in Fig. 7C and Supplementary Fig. S9A, iii–S9D, iii together with known oncogenic drivers and genes associated with ovarian cancer that are contained within the emergent resistant‐specific CNAs. The genomic regions at which copy number changes were observed and called by the LiqCNA algorithm differed between patients.

**Figure 8.**
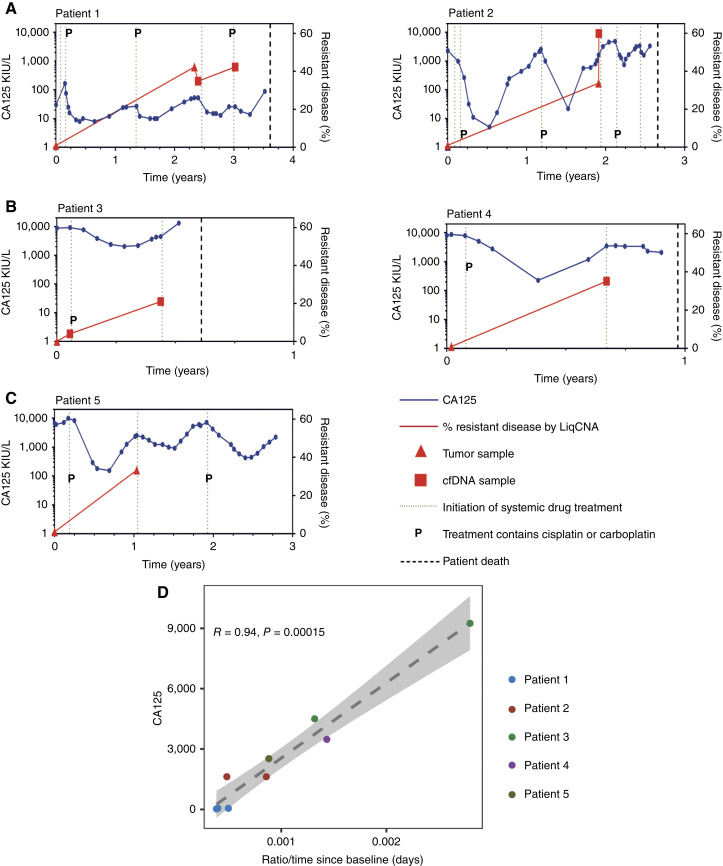
LiqCNA estimates of the emergent resistant population correlate with disease burder. **A–C,** LiqCNA estimation of resistant disease (percentage, red; right *y*‐axis) and CA125 (kIU/L, blue; left *y*‐axis) plotted over time for five unique patients. Time 0 = diagnosis. Subclonal ratio of tumor and blood samples is shown by triangles and squares, respectively. Dotted line indicates the start of each new line of chemotherapy, P, platinum‐containing chemotherapy; dashed line, date of patient death. **D,** Rate of change in the subclonal ratio estimated by LiqCNA in each sequential time point (compared with the diagnostic sample for that patient) plotted against CA125 at the later time point.

We then compared the change in the emergent resistant population calculated by LiqCNA with the change in CA125 for each patient over time (Fig. 8). These patients presented different manifestations of HGSC, with two showing a typical relapsing/remitting disease course (Patients 1 and 2; Fig. 8A), two showing minimal platinum sensitivity and poor survival (Patients 3 and 4; Fig. 8B), and one (Patients 5; Fig. 8C) demonstrating an intermediate disease course with repeated drug response but persistent high-volume disease. Clinical details are provided in Supplementary Table S2. We found a strong correlation (*R* = 0.94, *P* = 0.00015) between the inferred growth rate of the emergent resistant population in two sequential samples and the absolute CA125 at the time of the later LiqCNA estimation (Fig. 8D). LiqCNA measurements of resistant population growth therefore correlated strongly with disease burden. Moreover, in the three patients with LiqCNA readings at three time points (Patients 1, 2, and 3), the higher LiqCNA reading was followed by disease progression or a shorter time to next treatment. Together, this implies that LiqCNA could potentially provide a circulating marker to track platinum resistance and guide dose modulations in future AT clinical trials.

## Discussion

AT is based on the premise that drug-resistant phenotypes have reduced fitness in resource‐limited environments, because of the cellular resources they require to evade therapy (5, 8). Although this may only represent a small proportion of energy expenditure, in the resource‐poor conditions typical within the tumor microenvironment, these small changes are sufficient to impair cellular processes (28). Fitness penalties have been demonstrated in BRAF-resistant melanoma (29) and EGFR-resistant metastatic colorectal cancer (30) but have not been defined during the evolution of resistance to common cytotoxic therapies like platinum chemotherapy. However, the association between fitness and drug resistance is not inevitable (28, 31, 32) and loss of fitness is not essential for AT to be effective (10). Conversely, fitness loss does not guarantee AT efficacy as alternative mechanisms, for example, ecological interactions (bioRxiv 2023.03.16.533001) and spatial constraints (33), may compensate for resistance-associated fitness deficits. We observed competition *in vitro* when serum concentration was 20-fold lower than standard culture conditions. Other alterations could plausibly affect cell growth and others have observed fitness deficits during hypoxia in colorectal cancer (33) and low glucose in colorectal (33) and breast cancers (34).

We recognize the limitations of inducing resistance via prolonged drug exposure *in vitro* but mitigate this via our IVR01 cells, in which resistance was evolved in an intraperitoneal *in vivo* model that more accurately represents the clinical situation (20). We observed costs of resistance in all cell lines and *in vivo* models. Future work could determine whether these features are stable in repeated rounds of evolution. We provide compelling evidence in support of AT, particularly in mice with sensitive tumors for which tumor control was significantly extended compared with standard carboplatin dosing, with only one mouse experiencing disease progression. Interestingly, in mice with resistant tumors, standard carboplatin accelerated tumor growth. This could be explained by the phenomenon of “competitive release,” in which high drug dose may have eliminated any remaining sensitive cells within these comparatively resistant tumors, facilitating growth of the resistant cells, which made up the bulk of the tumor (35).

Our AT regimen reduced carboplatin dose rapidly as tumors shrank so that by the end of the experiment, these mice were receiving repeated, very small carboplatin doses. This resulted in higher cumulative drug dose compared with ST. A similar pattern has been seen in other preclinical AT studies in ovary (18) and breast cancers (27). Our experiment could not determine whether the extended survival with AT was due to evolutionary factors or simply due to the higher cumulative drug dose, although we note that AT did not increase mean daily carboplatin dose. Others have shown that repeated administration of standard therapy results in worse survival compared with AT, implying that the dynamics of AT dosing rather than drug dosage are responsible for the clinical benefit observed (27).

We were encouraged to note that the survival benefit we observed with AT was achieved without excess toxicity. In contrast, a recent article testing AT attempted to achieve dose equivalence by administering continuous MTD chemotherapy to control-treated mice (36). This frequently resulted in worse survival, likely because of excess toxicity. In humans, tolerability limits the number of chemotherapy doses and so chemotherapy is usually given as a course of six treatments followed by a treatment break until the next clinical relapse. Trials have shown that prolonged courses of high-dose chemotherapy do not result in additional clinical benefit (37). There is no standard carboplatin regimen for mice; hence, we used the same ST regimen as Gatenby and colleagues (18). In our experiment, this regimen only achieved tumor stabilization, rather than shrinkage. This implies that even our OVCAR4 tumors had limited carboplatin sensitivity and highlights the pronounced benefit of carboplatin AT in this model. We recognize that we could have included greater animal numbers and additional cohorts treated with different dosing regimens. However, clinical data are currently being generated via the multicenter, randomized ACTOv clinical trial (Adaptive ChemoTherapy in Ovarian Cancer; refs. 38, 39), comparing a related carboplatin AT regimen (optimized for human patients) to standard dosing in women with heavily pretreated, platinum-resistant ovarian cancer.

AT is expected to be most effective when dosing regimens respond to evolving tumor dynamics. The phase II CHRONOS trial in metastatic colorectal cancer provided proof of principle that cfDNA could guide therapeutic rechallenge with panitumumab (40), and the DYNAMIC trial will use ctDNA to direct AT with BRAF and MEK inhibitors in advanced malignant melanoma (41). Carboplatin resistance is not associated with recurrent point mutations or copy number changes (19, 21, 22) and we confirm this in our patient data. However, we observed passenger CNAs that are specific to individual patients and showed that LiqCNA can use these changes to measure the emergence of resistance in individual patients with ovarian cancer. Although LiqCNA does not shed light on the cost of resistance, it correlated strongly with tumor growth in our small patient cohort.

In summary, we have shown that AT is significantly more effective in HGSC than standard carboplatin dosing, achieving long‐term tumor control. Together, our findings that less fit resistant populations decline by reduced proliferation and increased apoptosis, that sensitive/resistant populations fluctuate through therapy, and that AT is not associated with increased drug dose per day, strongly imply that the success we observed with AT is indeed due to differences in relative population fitness as drug is applied and withdrawn. LiqCNA is a new way to measure the emergent resistant population in cfDNA, and ACTOv will further validate LiqCNA in up to 15 sequential samples per patient. This is expected to enhance our understanding of clonal evolution in HGSC during carboplatin therapy and potentially lead to the use of LiqCNA as a biomarker to direct AT in second-generation clinical trials.

## Supplementary Material

Supplementary Table 1 List of cell line names, platinum sensitivity and method used to evolve drug resistance (in vitro or in vivo).

Supplementary Table 2 Tissue and blood samples were collected from 5 patients. Patient age, stage of disease at diagnosis, BRCA mutation status and timings of tissue and blood samples obtained are shown. BRCA1m = mutation in BRCA1 gene, WT = wild type).

Supplementary Figure 1 A: Subcutaneous xenografts of OVCAR4 (black) and Ov4Carbo (orange) in both flanks of female CD1nu/nu mice measured with callipers over time. (n=3 mice per cell line). B: Pre-treatment growth of tumours shown in Fig.4B. Size criteria for inclusion in the study is indicated by the dotted line at 300mm3

Supplementary Figure 2 Cumulative dose of carboplatin in mg/kg over time for all mice receiving adaptive therapy. Green=100% sensitive, blue=80% sensitive, red=100% resistant with a different shade for each mouse. A-C: Cumulative carboplatin (mg) plotted against volume of individual tumours separated into A: OVCAR4, sensitive, green, B: 80:20 OVCAR4:Ov4Carbo-Luc, blue and C: Ov4Carbo-Luc, resistant, red. Each line indicates one tumour and the same colour shade is used for the same mouse in Fig.2C. D: Carboplatin dose per day according to injected cell ratio and treatment group. mean±st.d, n=2‐5 mice per group. *p<0.05, paired t‐test.

Supplementary Figure 3 A: Animal weight over time for mice enrolled in the experiment shown in Fig.4B, using the same colour codes. *= AT-treated OVCAR4 mouse that was culled before experimental end point due to unexplained weight loss with no tumour seen at necropsy. B: Mean animal weight over time according to injected cell type: green=OVCAR4, blue=80:20 OVCAR4:Ov4Carbo, red=Ov4Carbo-Luc, and treatment group: triangle = vehicle, circle=ST and square=AT. n=2-5 per group.

Supplementary Figure 4 Cells were seeded in 10% FBS-containing media and after 24 hours, media was changed to either 10% or 0.5% FBS-containing media. Media was exchanged for new media with the same FBS concentration every 24 hours. Cells were imaged every four hours for 120 hours with a 10x incucyte brightfield microscope to measure % confluence. mean±s.d., N=4 biological repeat experiments.

Supplementary Figure 5 A: Doxorubicin-induced expression of p16 and p21 in OVCAR4 and Ov4Cis cells by reverse transcription qPCR (mean±st.d, n=3, **p<0.01, unpaired t-test). B: OVCAR4 and Ov4Cis cells were grown as mono-culture and as 85:15 S:R and 50:50 S:R co-cultures. Co-culture samples were sorted into GFP-positive and GFP-negative populations by flow cytometry. Expression of p16 and C: p21 by reverse transcription qPCR in each population is shown (mean±st.d, n=3). Horizontal dotted lines indicate a 2-fold and 0.5-fold increase in gene expression.

Supplementary Figure 6 Mean fluorescence of OVCAR4 (GFP positive) and Ov4Cis (RFP positive) for up to 20 passages in vitro (mean±st.d, n=3 technical replicates).

Supplementary Figure 7 Quantitative PCR measures of GFP and RFP DNA obtained from co-cultures of OVCAR4 (GFP positive) and Ov4Cis (RFP positive) cells at a range of ratios (100:0, 85:15, 75:25, 50:50, 25:75, 15:85, 0:100). Calculated 2^(-ddCT)x100 values are plotted against the known starting input ratio of OVCAR4 (i.) and Ov4Cis (ii).

Supplementary Figure 8 Mice were inoculated subcutaneously with OVCAR4 and Ov4Cis cells at 50:50 or 80:20 and culled over time. GFP and RFP DNA was measured by qPCR in each tumour and normalised to total tumour DNA (hGAPDH) and 100% GFP and 100% RFP tumours (mean±st.d, n=4 tumours per condition).

Supplementary Figure 9 A-Di: Tumour purity-corrected copy number profile for each sample obtained from patients UP0018 (A), UP0042 (B), UP0053 (C) and UP0056 (D). Grey bars show the copy number of each segment in the baseline diagnostic tumour biopsy sample and red bars show CN profile of later samples as indicated. A-Dii: Resistant proportion of each sample estimated by LiqCNA. Error bars indicate 95% confidence of each estimate. A-Diii: Zoomed-in profiles of selected chromosomes with the most prominent/impactful resistant-specific copy number alterations. Driver genes or genes associated with ovarian cancer that overlap with resistant-specific CNA are indicated by blue vertical lines and listed below each graph.
