# Supplementary material for: Adaptive Therapy Exploits Fitness Deficits in Chemotherapy-Resistant Ovarian Cancer to Achieve Long-Term Tumor Control
Source: Cancer Res. 2025 Apr 29;85(18):3503–17. doi: 10.1158/0008-5472.CAN-25-0351 (PMC12434395; doi:10.1158/0008-5472.CAN-25-0351)
Supplement: Supplementary Table 1 — List of cell line names, platinum sensitivity and method used to evolve drug resistance (in vitro or in vivo). [file can-25-0351_supplementary_table_1_suppst1.docx]

| **Name** | **Evolved resistant (Y/N)** | **Ancestral cell line** | **Drug used to evolve resistance** | **Resistance evolved *in vitro/in vivo*** |
| --- | --- | --- | --- | --- |
| **OVCAR4** | N | - | - | - |
| **Ov4Carbo** | Y | OVCAR4 | Carboplatin | *In vitro* |
| **Ov4Cis** | Y | OVCAR4 | Cisplatin | *In vitro* |
| **IVR01** | Y | OVCAR4 | Carboplatin | *In vivo* |
| **Cov318** | N | - | -- | *-* |
| **Cov-Cis** | Y | Cov318 | Cisplatin | *In vitro* |

**Supplementary Table 1**

List of cell line names, platinum sensitivity and method used to evolve drug resistance (*in vitro* or *in vivo).*
