# Supplementary material for: Adaptive Therapy Exploits Fitness Deficits in Chemotherapy-Resistant Ovarian Cancer to Achieve Long-Term Tumor Control
Source: Cancer Res. 2025 Apr 29;85(18):3503–17. doi: 10.1158/0008-5472.CAN-25-0351 (PMC12434395; doi:10.1158/0008-5472.CAN-25-0351)
Supplement: Supplementary Table 2 — Tissue and blood samples were collected from 5 patients. Patient age, stage of disease at diagnosis, BRCA mutation status and timings of tissue and blood samples obtained are shown. BRCA1m = mutation in BRCA1 gene, WT = wild type). [file can-25-0351_supplementary_table_2_suppst2.docx]

**Supplementary Table** **2**

Tissue and blood samples were collected from 5 patients. Patient age, stage of disease at diagnosis, BRCA mutation status and timings of tissue and blood samples obtained are shown.

**
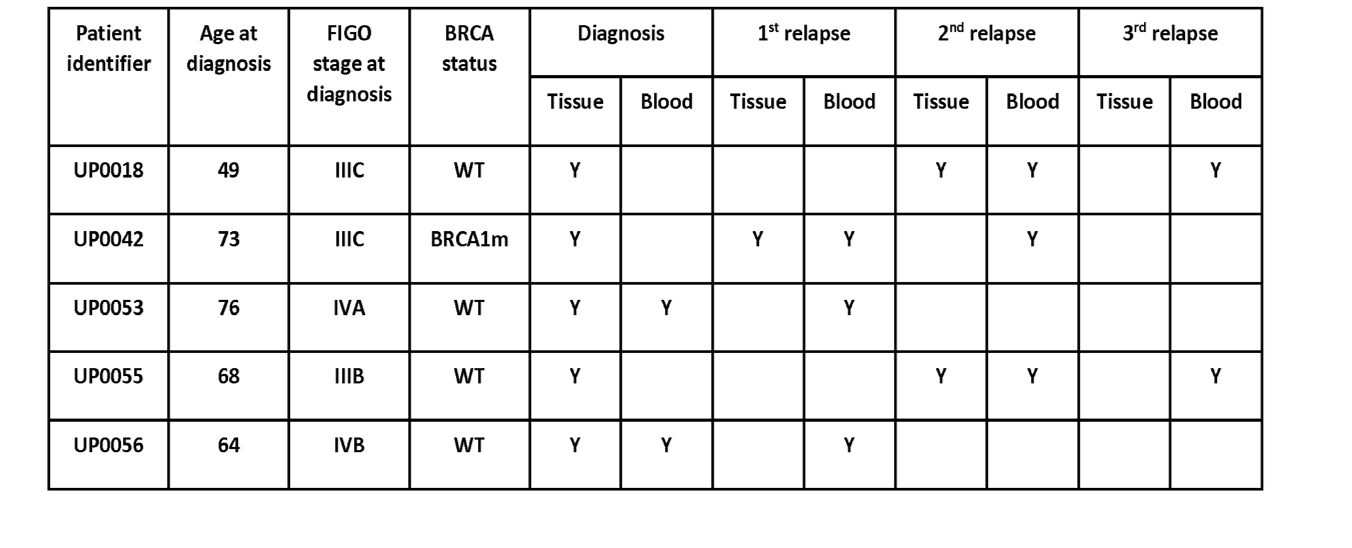
**(BRCA1m = mutation in BRCA1 gene, WT = wild type).
