# Supplementary material for: Adaptive Therapy Exploits Fitness Deficits in Chemotherapy-Resistant Ovarian Cancer to Achieve Long-Term Tumor Control
Source: Cancer Res. 2025 Apr 29;85(18):3503–17. doi: 10.1158/0008-5472.CAN-25-0351 (PMC12434395; doi:10.1158/0008-5472.CAN-25-0351)
Supplement: Supplementary Figure 1 — A: Subcutaneous xenografts of OVCAR4 (black) and Ov4Carbo (orange) in both flanks of female CD1nu/nu mice measured with callipers over time. (n=3 mice per cell line). B: Pre-treatment growth of tumours shown in Fig.4B. Size criteria for inclusion in the study is indicated by the dotted line at 300mm3 [file can-25-0351_supplementary_figure_1_suppsf1.pdf]

S1

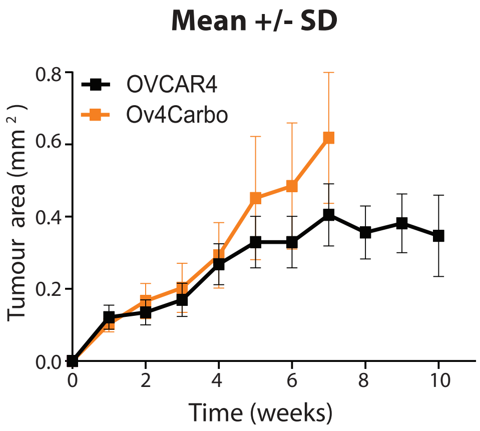

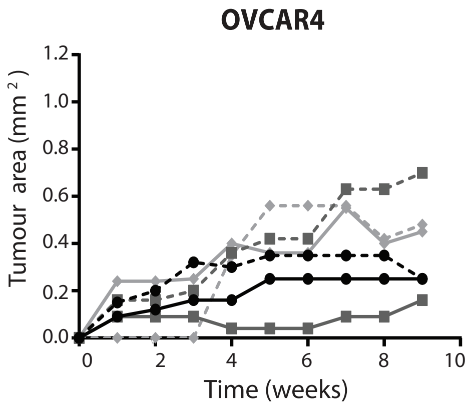

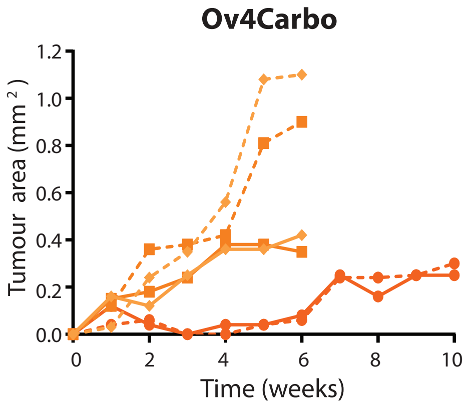

A: Subcutaneous xenografts of OVCAR4 (black) and Ov4Carbo (orange) in both flanks of female CD1nu/nu mice measured with callipers over time. (*n*=3 mice per cell line). **B**: Pre-treatment growth of tumours shown in Fig.4B. Size criteria for inclusion in the study is indicated by the dotted line at 300mm^3
