# Supplementary material for: Adaptive Therapy Exploits Fitness Deficits in Chemotherapy-Resistant Ovarian Cancer to Achieve Long-Term Tumor Control
Source: Cancer Res. 2025 Apr 29;85(18):3503–17. doi: 10.1158/0008-5472.CAN-25-0351 (PMC12434395; doi:10.1158/0008-5472.CAN-25-0351)
Supplement: Supplementary Figure 2 — Cumulative dose of carboplatin in mg/kg over time for all mice receiving adaptive therapy. Green=100% sensitive, blue=80% sensitive, red=100% resistant with a different shade for each mouse. A-C: Cumulative carboplatin (mg) plotted against volume of individual tumours separated into A: OVCAR4, sensitive, green, B: 80:20 OVCAR4:Ov4Carbo-Luc, blue and C: Ov4Carbo-Luc, resistant, red. Each line indicates one tumour and the same colour shade is used for the same mouse in Fig.2C. D: Carboplatin dose per day according to injected cell ratio and treatment group. mean±st.d, n=2‐5 mice per group. *p<0.05, paired t‐test. [file can-25-0351_supplementary_figure_2_suppsf2.pdf]

S2

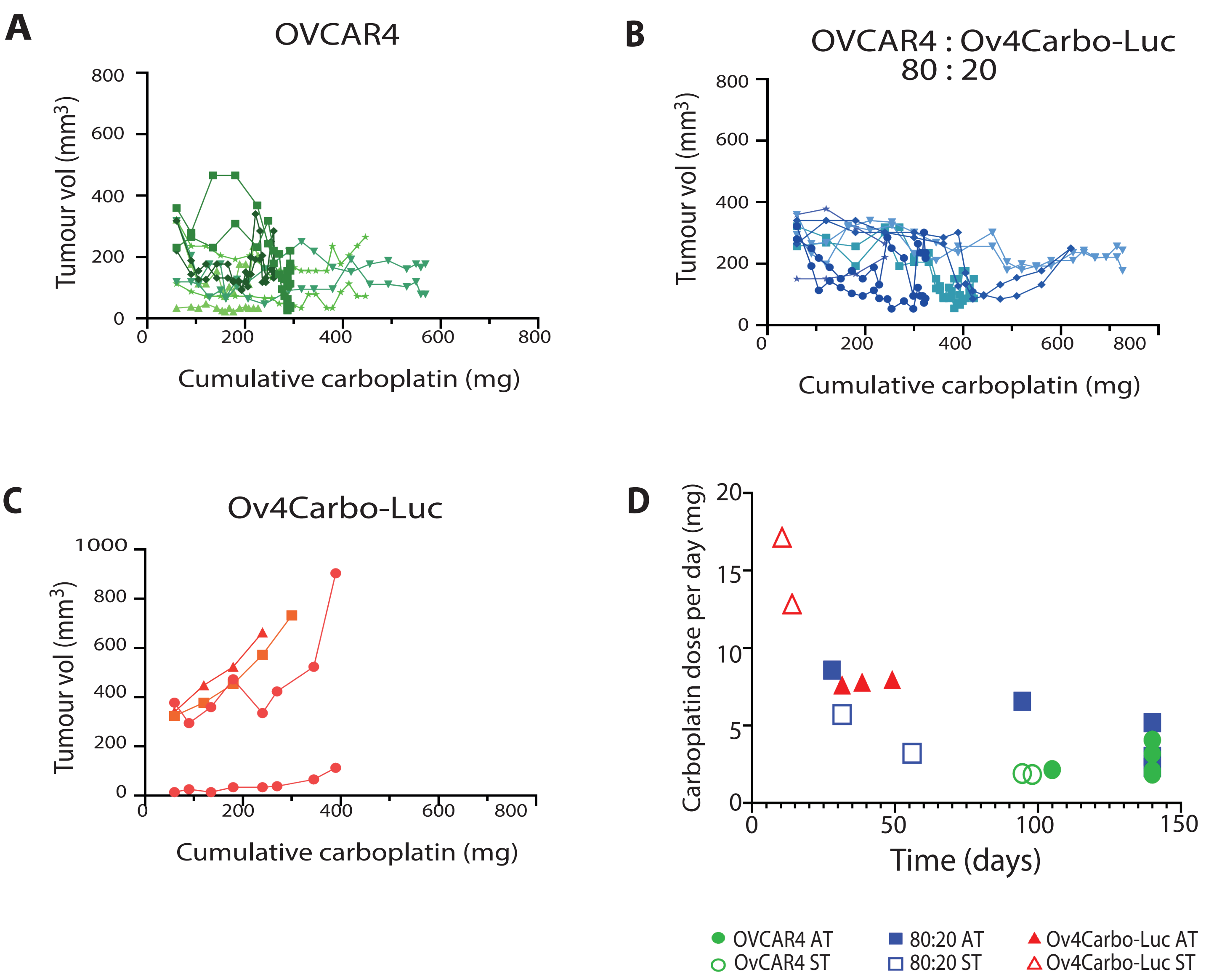

Cumulative dose of carboplatin in mg/kg over time for all mice receiving adaptive therapy. Green=100% sensitive, blue=80% sensitive, red=100% resistant with a different shade for each mouse. **A-C:** Cumulative carboplatin (mg) plotted against volume of individual tumours separated into **A:** OVCAR4, sensitive, green, **B:** 80:20 OVCAR4:Ov4Carbo-Luc, blue and **C:** Ov4Carbo-Luc, resistant, red. Each line indicates one tumour and the same colour shade is used for the same mouse in Fig.2C. **D:** Carboplatin dose per day according to injected cell ratio and treatment group. mean±st.d, n=2-5 mice per group. *$p<0.05$, paired t-test.
