# Supplementary material for: Adaptive Therapy Exploits Fitness Deficits in Chemotherapy-Resistant Ovarian Cancer to Achieve Long-Term Tumor Control
Source: Cancer Res. 2025 Apr 29;85(18):3503–17. doi: 10.1158/0008-5472.CAN-25-0351 (PMC12434395; doi:10.1158/0008-5472.CAN-25-0351)
Supplement: Supplementary Figure 3 — A: Animal weight over time for mice enrolled in the experiment shown in Fig.4B, using the same colour codes. *= AT-treated OVCAR4 mouse that was culled before experimental end point due to unexplained weight loss with no tumour seen at necropsy. B: Mean animal weight over time according to injected cell type: green=OVCAR4, blue=80:20 OVCAR4:Ov4Carbo, red=Ov4Carbo-Luc, and treatment group: triangle = vehicle, circle=ST and square=AT. n=2-5 per group. [file can-25-0351_supplementary_figure_3_suppsf3.pdf]

S3

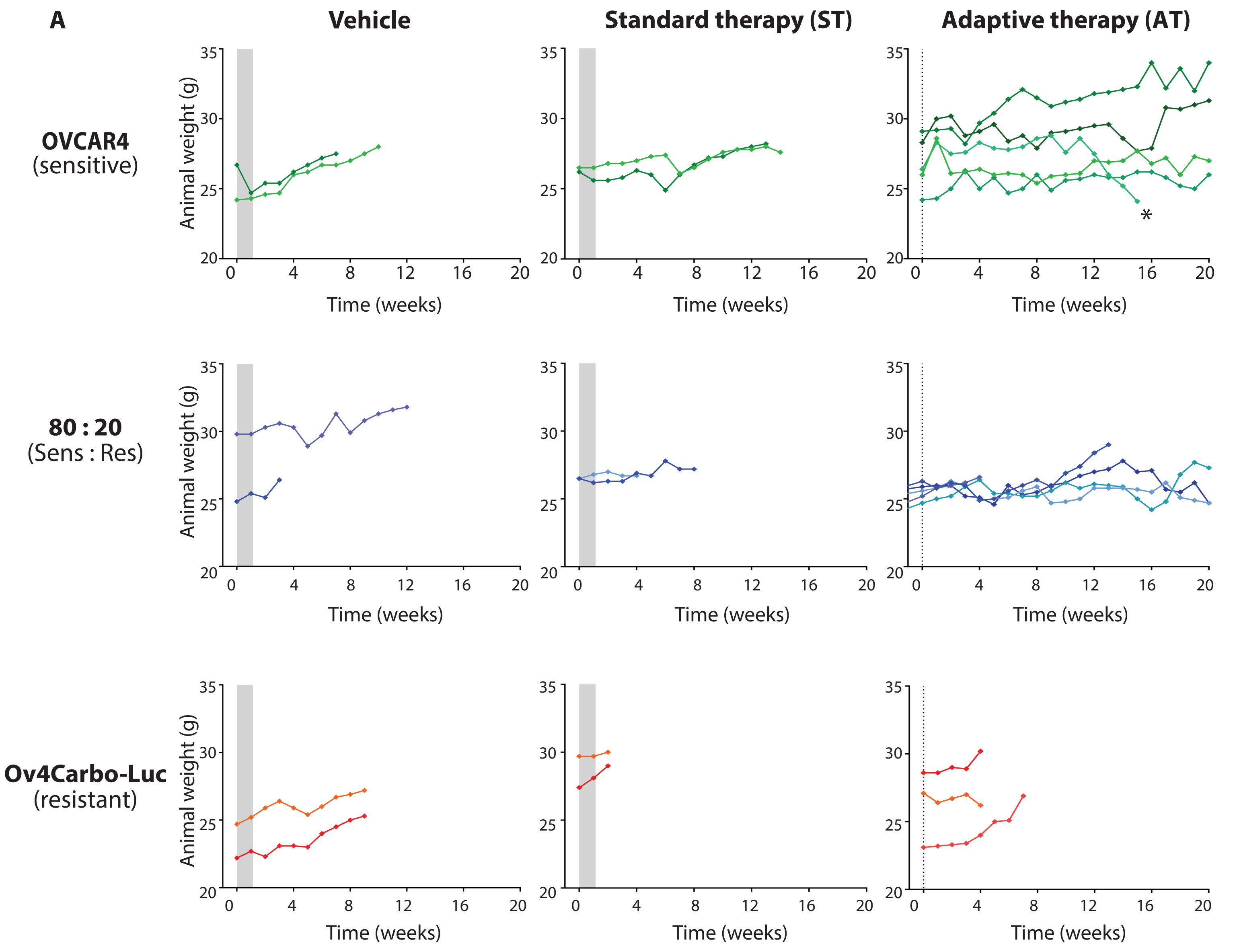

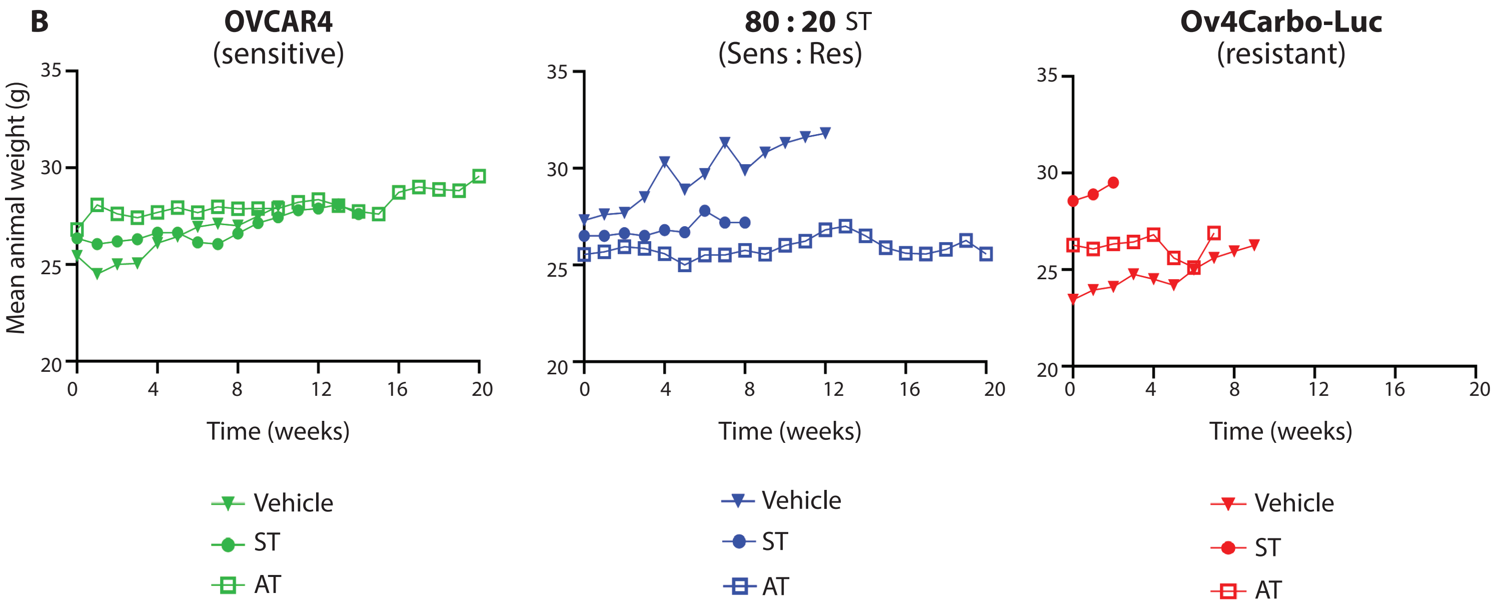

A: Animal weight over time for mice enrolled in the experiment shown in Fig.4B, using the same colour codes. *= AT-treated OVCAR4 mouse that was culled before experimental end point due to unexplained weight loss with no tumour seen at necropsy. **B:** Mean animal weight over time according to injected cell type: green=OVCAR4, blue=80:20 OVCAR4:Ov4Carbo, red=Ov4Carbo-Luc, and treatment group: triangle = vehicle, circle=ST and square=AT. *n*=2-5 per group.
