# Supplementary material for: Adaptive Therapy Exploits Fitness Deficits in Chemotherapy-Resistant Ovarian Cancer to Achieve Long-Term Tumor Control
Source: Cancer Res. 2025 Apr 29;85(18):3503–17. doi: 10.1158/0008-5472.CAN-25-0351 (PMC12434395; doi:10.1158/0008-5472.CAN-25-0351)
Supplement: Supplementary Figure 4 — Cells were seeded in 10% FBS-containing media and after 24 hours, media was changed to either 10% or 0.5% FBS-containing media. Media was exchanged for new media with the same FBS concentration every 24 hours. Cells were imaged every four hours for 120 hours with a 10x incucyte brightfield microscope to measure % confluence. mean±s.d., N=4 biological repeat experiments. [file can-25-0351_supplementary_figure_4_suppsf4.pdf]

S4

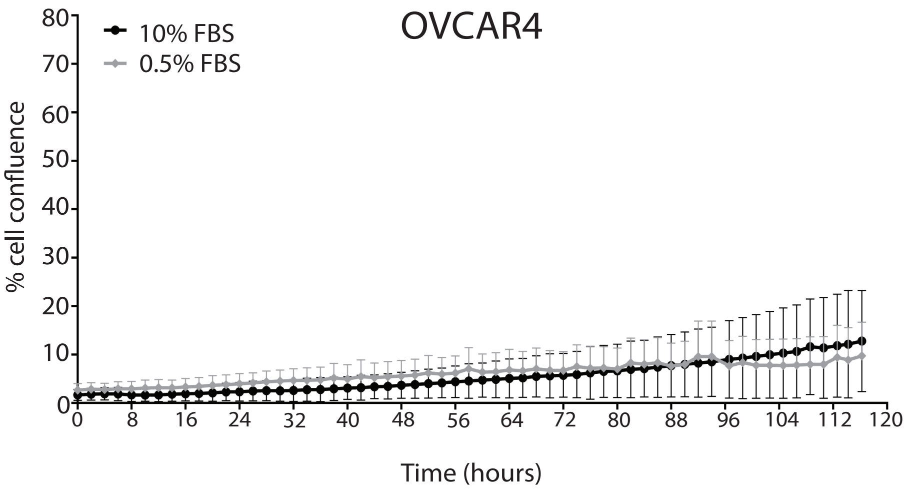

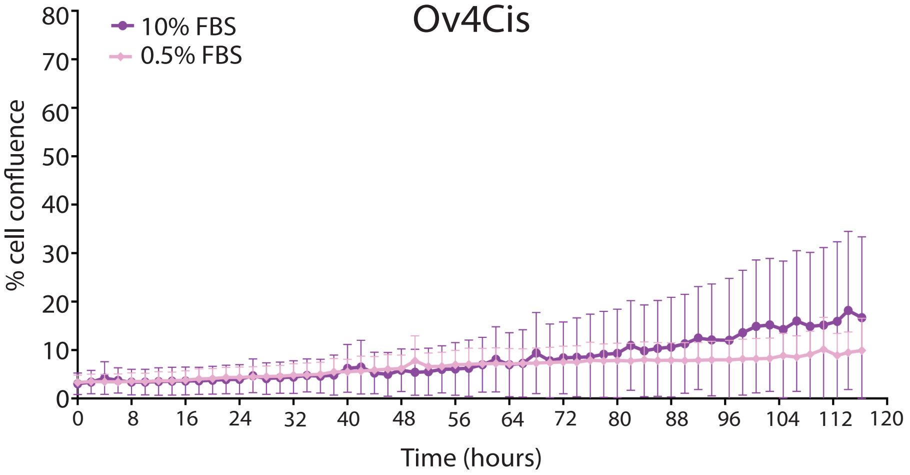

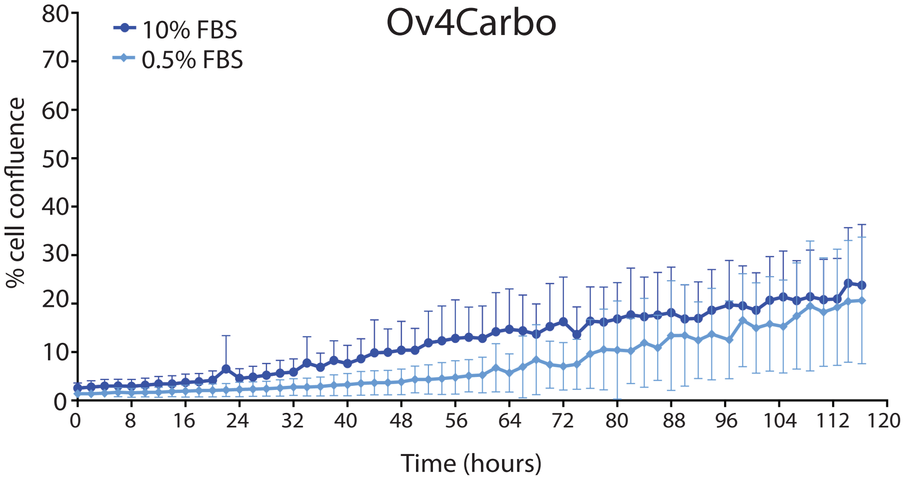

Cells were seeded in 10% FBS-containing media and after 24 hours, media was changed to either 10% or 0.5% FBS-containing media. Media was exchanged for new media with the same FBS concentration every 24 hours. Cells were imaged every four hours for 120 hours with a 10x incucyte® brightfield microscope to measure % confluence. mean±s.d., *N*=4 biological repeat experiments.
