# Supplementary material for: Adaptive Therapy Exploits Fitness Deficits in Chemotherapy-Resistant Ovarian Cancer to Achieve Long-Term Tumor Control
Source: Cancer Res. 2025 Apr 29;85(18):3503–17. doi: 10.1158/0008-5472.CAN-25-0351 (PMC12434395; doi:10.1158/0008-5472.CAN-25-0351)
Supplement: Supplementary Figure 5 — A: Doxorubicin-induced expression of p16 and p21 in OVCAR4 and Ov4Cis cells by reverse transcription qPCR (mean±st.d, n=3, **p<0.01, unpaired t-test). B: OVCAR4 and Ov4Cis cells were grown as mono-culture and as 85:15 S:R and 50:50 S:R co-cultures. Co-culture samples were sorted into GFP-positive and GFP-negative populations by flow cytometry. Expression of p16 and C: p21 by reverse transcription qPCR in each population is shown (mean±st.d, n=3). Horizontal dotted lines indicate a 2-fold and 0.5-fold increase in gene expression. [file can-25-0351_supplementary_figure_5_suppsf5.pdf]

S5

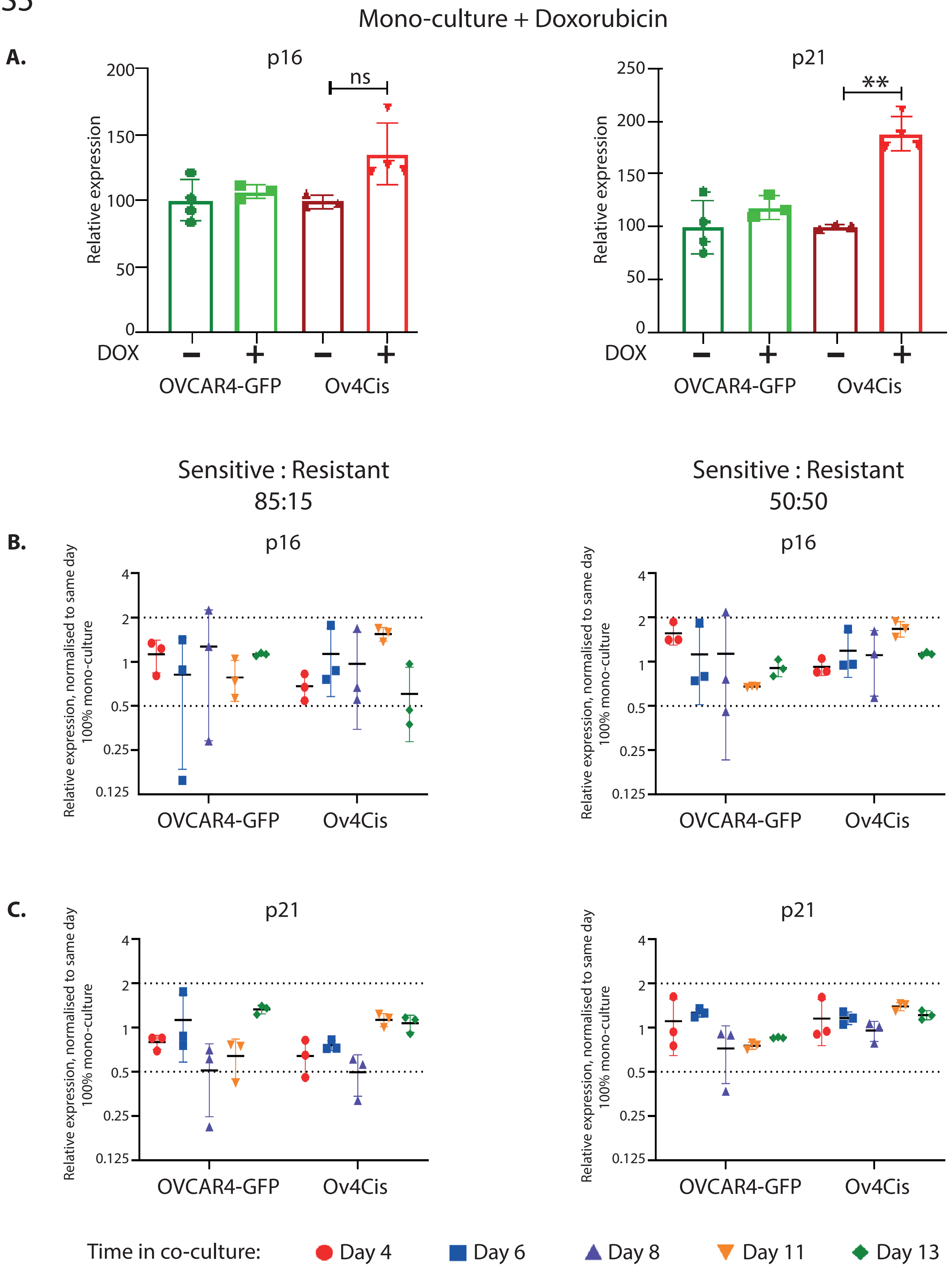

A: Doxorubicin-induced expression of p16 and p21 in OVCAR4 and Ov4Cis cells by reverse transcription qPCR (mean±st.d, *n*=3, **p<0.01, unpaired *t*-test). **B:** OVCAR4 and Ov4Cis cells were grown as mono-culture and as 85:15 S:R and 50:50 S:R co-cultures. Co-culture samples were sorted into GFP-positive and GFP-negative populations by flow cytometry. Expression of p16 and **C:** p21 by reverse transcription qPCR in each population is shown (mean±st.d, *n*=3). Horizontal dotted lines indicate a 2-fold and 0.5-fold increase in gene expression.
