# Supplementary material for: Adaptive Therapy Exploits Fitness Deficits in Chemotherapy-Resistant Ovarian Cancer to Achieve Long-Term Tumor Control
Source: Cancer Res. 2025 Apr 29;85(18):3503–17. doi: 10.1158/0008-5472.CAN-25-0351 (PMC12434395; doi:10.1158/0008-5472.CAN-25-0351)
Supplement: Supplementary Figure 6 — Mean fluorescence of OVCAR4 (GFP positive) and Ov4Cis (RFP positive) for up to 20 passages in vitro (mean±st.d, n=3 technical replicates). [file can-25-0351_supplementary_figure_6_suppsf6.pdf]

S6

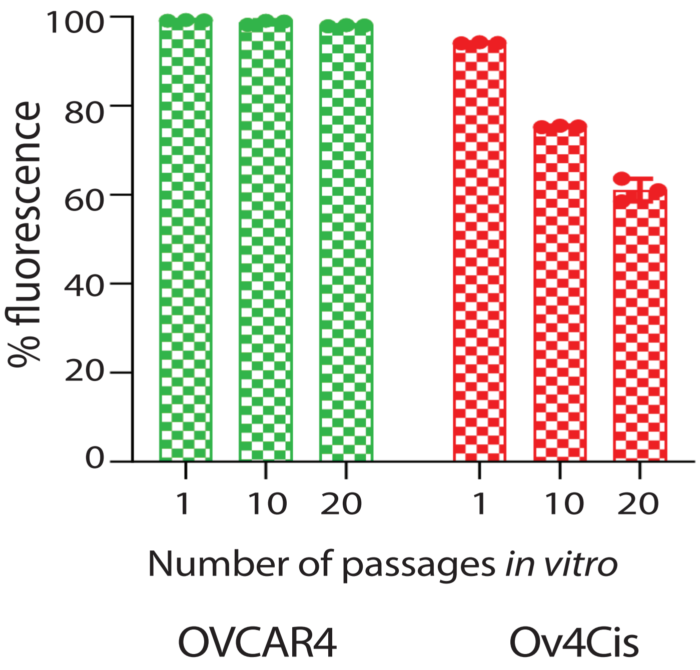

Mean fluorescence of OVCAR4 (GFP positive) and Ov4Cis (RFP positive) for up to 20 passages *in vitro* (mean±st.d, *n*=3 technical replicates).
