# Supplementary material for: Adaptive Therapy Exploits Fitness Deficits in Chemotherapy-Resistant Ovarian Cancer to Achieve Long-Term Tumor Control
Source: Cancer Res. 2025 Apr 29;85(18):3503–17. doi: 10.1158/0008-5472.CAN-25-0351 (PMC12434395; doi:10.1158/0008-5472.CAN-25-0351)
Supplement: Supplementary Figure 7 — Quantitative PCR measures of GFP and RFP DNA obtained from co-cultures of OVCAR4 (GFP positive) and Ov4Cis (RFP positive) cells at a range of ratios (100:0, 85:15, 75:25, 50:50, 25:75, 15:85, 0:100). Calculated 2^(-ddCT)x100 values are plotted against the known starting input ratio of OVCAR4 (i.) and Ov4Cis (ii). [file can-25-0351_supplementary_figure_7_suppsf7.pdf]

S7

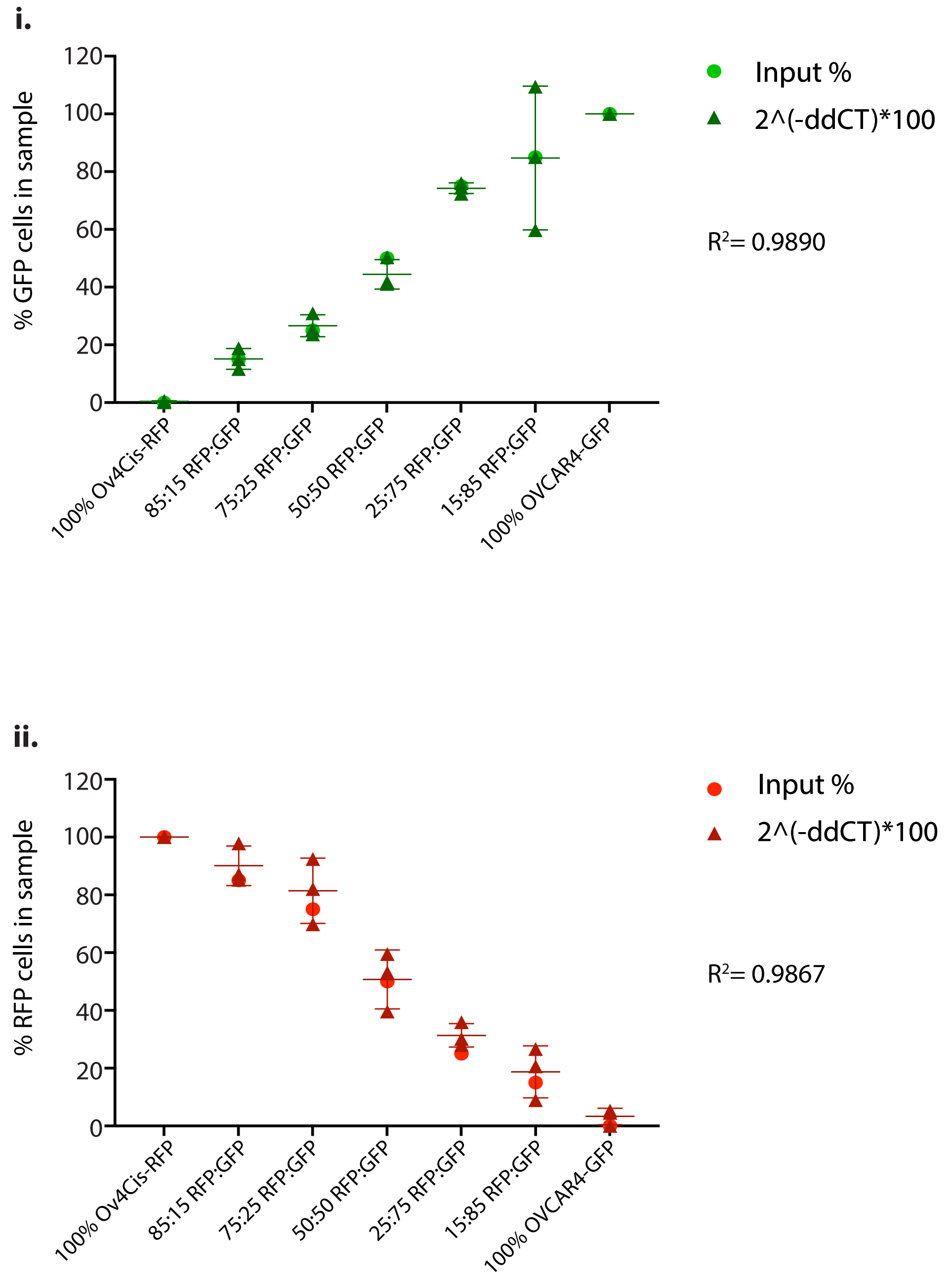

Quantitative PCR measures of GFP and RFP DNA obtained from co-cultures of OVCAR4 (GFP positive) and Ov4Cis (RFP positive) cells at a range of ratios (100:0, 85:15, 75:25, 50:50, 25:75, 15:85, 0:100). Calculated 2^(-ddCT)x100 values are plotted against the known starting input ratio of OVCAR4 (**i.**) and Ov4Cis (**ii**).
