# Supplementary material for: Adaptive Therapy Exploits Fitness Deficits in Chemotherapy-Resistant Ovarian Cancer to Achieve Long-Term Tumor Control
Source: Cancer Res. 2025 Apr 29;85(18):3503–17. doi: 10.1158/0008-5472.CAN-25-0351 (PMC12434395; doi:10.1158/0008-5472.CAN-25-0351)
Supplement: Supplementary Figure 8 — Mice were inoculated subcutaneously with OVCAR4 and Ov4Cis cells at 50:50 or 80:20 and culled over time. GFP and RFP DNA was measured by qPCR in each tumour and normalised to total tumour DNA (hGAPDH) and 100% GFP and 100% RFP tumours (mean±st.d, n=4 tumours per condition). [file can-25-0351_supplementary_figure_8_suppsf8.pdf]

S8

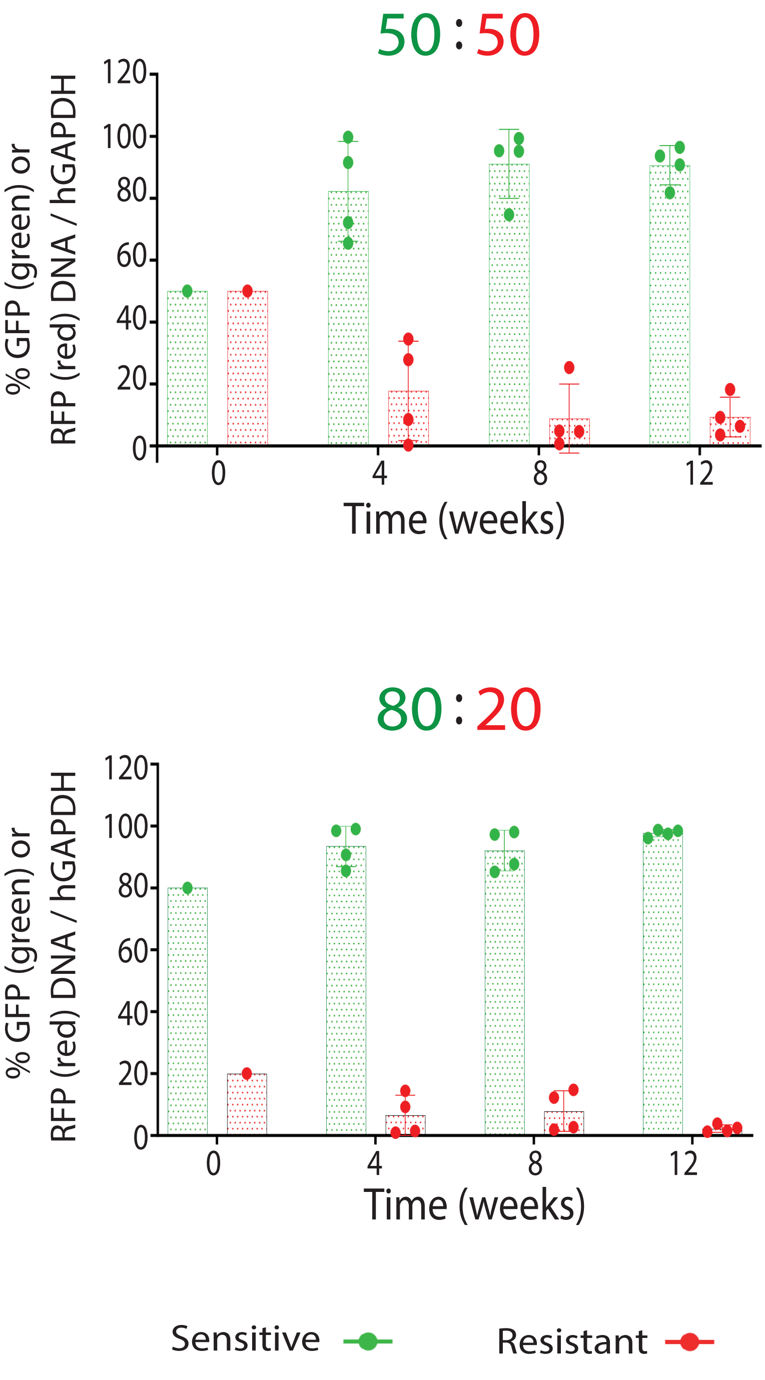

Mice were inoculated subcutaneously with OVCAR4 and Ov4Cis cells at 50:50 or 80:20 and culled over time. GFP and RFP DNA was measured by qPCR in each tumour and normalised to total tumour DNA (hGAPDH) and 100% GFP and 100% RFP tumours (mean±st.d, *n*=4 tumours per condition).
