# Supplementary material for: Adaptive Therapy Exploits Fitness Deficits in Chemotherapy-Resistant Ovarian Cancer to Achieve Long-Term Tumor Control
Source: Cancer Res. 2025 Apr 29;85(18):3503–17. doi: 10.1158/0008-5472.CAN-25-0351 (PMC12434395; doi:10.1158/0008-5472.CAN-25-0351)
Supplement: Supplementary Figure 9 — A-Di: Tumour purity-corrected copy number profile for each sample obtained from patients UP0018 (A), UP0042 (B), UP0053 (C) and UP0056 (D). Grey bars show the copy number of each segment in the baseline diagnostic tumour biopsy sample and red bars show CN profile of later samples as indicated. A-Dii: Resistant proportion of each sample estimated by LiqCNA. Error bars indicate 95% confidence of each estimate. A-Diii: Zoomed-in profiles of selected chromosomes with the most prominent/impactful resistant-specific copy number alterations. Driver genes or genes associated with ovarian cancer that overlap with resistant-specific CNA are indicated by blue vertical lines and listed below each graph. [file can-25-0351_supplementary_figure_9_suppsf9.pdf]

S9

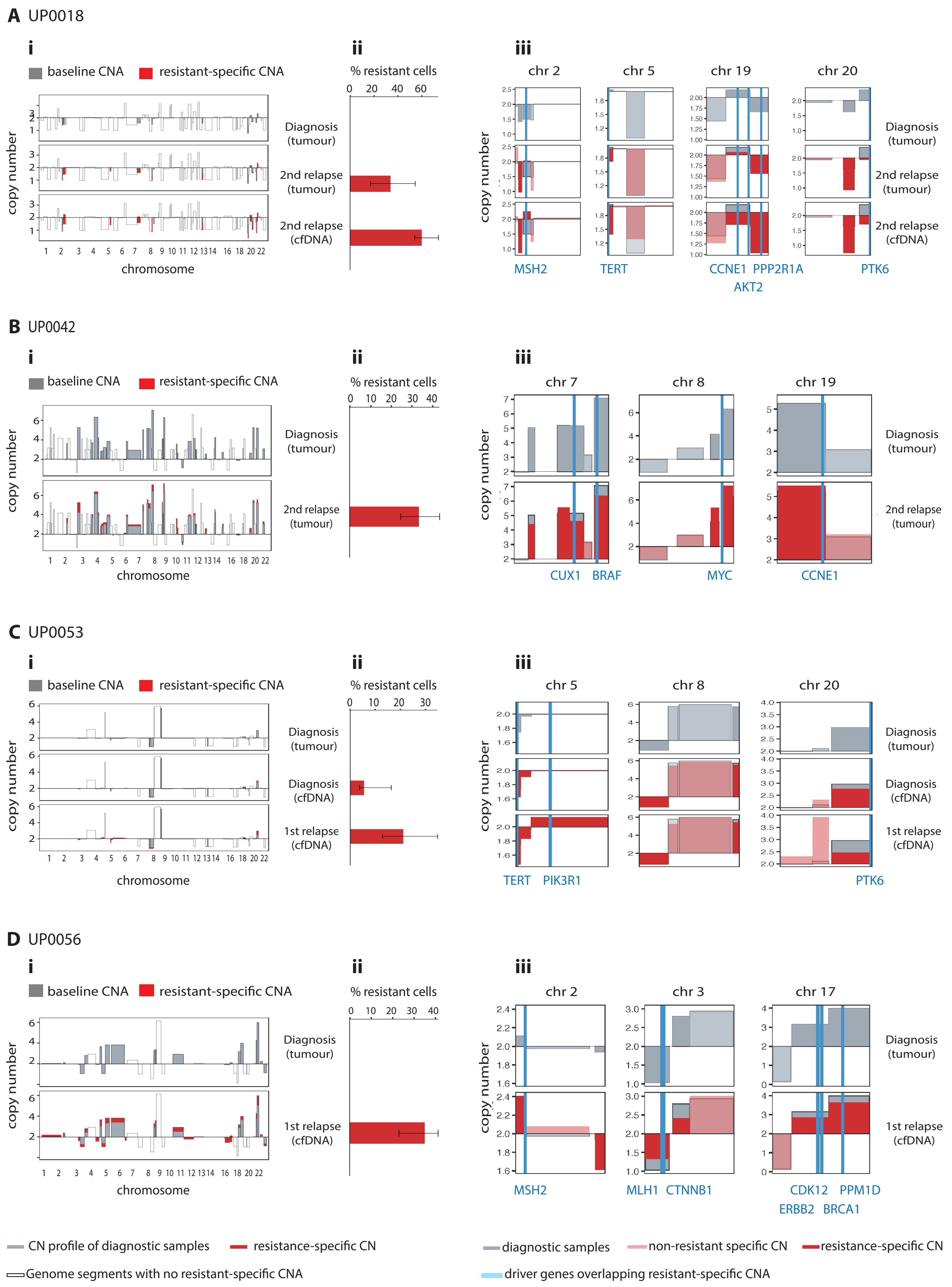
A UP0018
i
baseline CNA
resistant-specific CNA
copy number
chromosome
ii
% resistant cells
0 20 40 60
Diagnosis (tumour)
2nd relapse (tumour)
2nd relapse (cfDNA)
iii
chr 2
chr 5
chr 19
chr 20
MSH2
TERT
CCNE1 PPP2R1A
AKT2
PTK6
B UP0042
i
baseline CNA
resistant-specific CNA
copy number
chromosome
ii
% resistant cells
0 10 20 30 40
Diagnosis (tumour)
2nd relapse (tumour)
iii
chr 7
chr 8
chr 19
CUX1 BRAF
MYC
CCNE1
C UP0053
i
baseline CNA
resistant-specific CNA
copy number
chromosome
ii
% resistant cells
0 10 20 30
Diagnosis (tumour)
Diagnosis (cfDNA)
1st relapse (cfDNA)
iii
chr 5
chr 8
chr 20
TERT PIK3R1
PTK6
D UP0056
i
baseline CNA
resistant-specific CNA
copy number
chromosome
ii
% resistant cells
0 10 20 30 40
Diagnosis (tumour)
1st relapse (cfDNA)
iii
chr 2
chr 3
chr 17
MSH2
MLH1 CTNNB1
CDK12 PPM1D
ERBB2 BRCA1
CN profile of diagnostic samples
resistance-specific CN
Genome segments with no resistant-specific CNA
diagnostic samples
non-resistant specific CN
resistance-specific CN
driver genes overlapping resistant-specific CNA

A-Di: Tumour purity-corrected copy number profile for each sample obtained from patients UP0018 (**A**), UP0042 (**B**), UP0053 (**C**) and UP0056 (**D**). Grey bars show the copy number of each segment in the baseline diagnostic tumour biopsy sample and red bars show CN profile of later samples as indicated. **A-Dii:** Resistant proportion of each sample estimated by LiqCNA. Error bars indicate 95% confidence of each estimate. **A-Diii:** Zoomed-in profiles of selected chromosomes with the most prominent/impactful resistant-specific copy number alterations. Driver genes or genes associated with ovarian cancer that overlap with resistant-specific CNA are indicated by blue vertical lines and listed below each graph.
